# Characterization of the spectrum of trivalent *VAV1*‐mutation‐driven tumours using a gene‐edited mouse model

**DOI:** 10.1002/1878-0261.13295

**Published:** 2022-08-30

**Authors:** Javier Robles‐Valero, Lucía Fernández‐Nevado, Myriam Cuadrado, Luis Francisco Lorenzo‐Martín, Isabel Fernández‐Pisonero, Antonio Abad, Esther Redín, Luis Montuenga, Dionisio Martín‐Zanca, Anna Bigas, Moisés Mallo, Mercedes Dosil, Xosé R. Bustelo

**Affiliations:** ^1^ Molecular Mechanisms of Cancer Program, Centro de Investigación del Cáncer CSIC‐University of Salamanca Spain; ^2^ Instituto de Biología Molecular y Celular del Cáncer CSIC‐University of Salamanca Spain; ^3^ Centro de Investigación Biomédica en Red de Cáncer (CIBERONC) CSIC‐University of Salamanca Spain; ^4^ Solid Tumors Program, Center of Applied Medical Research University of Navarra Pamplona Spain; ^5^ Instituto de Biología Funcional y Genómica CSIC‐University of Salamanca Spain; ^6^ Institut Hospital del Mar d'Investigacions Médiques Barcelona Spain; ^7^ Gulbenkian Institute Oeiras Portugal

**Keywords:** angioimmunoblastic T cell lymphoma, follicular helper T cells, nonsmall‐cell lung cancer, peripheral T cell lymphoma, RAC1, TP53

## Abstract

Mutations in the VAV1 guanine nucleotide exchange factor 1 have been recently found in peripheral T cell lymphoma and nonsmall‐cell lung cancer (NSCLC). To understand their pathogenic potential, we generated a gene‐edited mouse model that expresses a VAV1 mutant protein that recapitulates the signalling alterations present in the *VAV1* mutant subclass most frequently found in tumours. We could not detect any overt tumourigenic process in those mice. However, the concurrent elimination of the *Trp53* tumour suppressor gene in them drives T cell lymphomagenesis. This process represents an exacerbation of the normal functions that wild‐type VAV1 plays in follicular helper T cells. We also found that, in combination with the *Kras* oncogene, the VAV1 mutant version favours progression of NSCLC. These data indicate that *VAV1* mutations play critical, although highly cell‐type‐specific, roles in tumourigenesis. They also indicate that such functions are contingent on the mutational landscape of the tumours involved.

## Graphical abstract

This work shows that the endogenous expression of an oncogenic version of the RHO exchange factor VAV1 (VAV1^Onc^) promotes T_FH_ cell hyperplasia and, upon loss of TP53, the generation of angioimmunoblastic T cell lymphoma. In addition, when coexpressed with KRAS^G12V^, it accelerates nonsmall‐cell lung cancer formation.

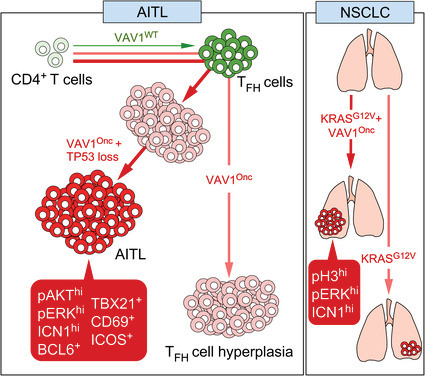

**Abbreviations**
AITL: angioimmunoblastic T cell lymphoma
ALCL: anaplastic large cell lymphoma
CBL‐B: E3 ubiquitin ligase Casitas B‐lineage lymphoma B
CC10: Clara cell‐specific protein
FDR: false discovery rate
GDP: guanosine diphosphate
GEO: gene expression omnibus
GSEA: gene set enrichment analysis
GTP: guanosine triphosphate
ICN1: intracellular fragment of Notch1
KLH: keyhole limpet haemocyanin
LSL‐*Kras* ^G12D/+^
: Lox‐Stop‐Lox‐ *Kras* ^G12D/+^
NES: normalized enrichment score
NFAT: nuclear factor of stimulated T cells
NP: nitrophenyl acetyl hapten
NSCLC: nonsmall‐cell lung cancer
NSt: nonstimulated
PLCγ1: phospholipase Cγ1
PTCL: peripheral T cell lymphoma
PTCL‐NOS: peripheral T cell lymphoma‐not otherwise specified
SEM: standard error of the mean
sgRNA: synthetic guide RNA
SPC: surfactant protein C
St: stimulated
TCR: T cell receptor
T_FH_ cells: follicular helper T cells
TOX: thymocyte selection associated high mobility group box
TP53‐ER^TAM^
: TP53‐estrogen receptor fusion protein regulated by tamoxifen

## 1 Introduction

VAV1 is a haematopoietic‐specific signalling protein that plays critical roles in the development, selection, and effector functions of T cells [1, 2, 3, 4]. This protein can engage multiple downstream programs using both catalysis‐dependent and independent pathways, thus contributing to the amplification and diversification of the signals that are initiated at the level of the T cell receptor (TCR) [1, 3]. The main function of this protein is to catalyse the exchange of nucleotides in RHO GTPases, thus favouring the transition of those proteins from the inactive (GDP‐bound) to the active (GTP‐bound) state [1, 3, 5]. This pathway is important for the regulation of cytoskeletal dynamics, the activation of downstream serine/threonine kinases (e.g., p21 activated and c‐Jun N‐terminal kinases), and the stimulation of F–actin‐regulated transcriptional programs [1, 3]. In addition, VAV1 plays several noncatalytic, adaptor‐like functions. Thus, via its C‐terminal calponin‐homology domain, VAV1 can engage a phospholipase Cγ1‐dependent signalling pathway that favours the stimulation of the nuclear factor of stimulated T cells (NFAT) [6, 7]. This pathway is critical for the stimulation of T cells, cytokine production, and differentiation of follicular helper T (T_FH_) cells [8, 9]. Finally, VAV1 regulates via its most C‐terminal SH3 (CSH3) domain an adaptor function that promotes the nucleation of heteromolecular complexes with the E3 ubiquitin ligase Casitas B‐lineage lymphoma B (CBL‐B) and the intracellular fragment of Notch1 (ICN1). This interaction favours the CBL‐B‐dependent ubiquitinylation and proteosomal degradation of ICN1, a key protumourigenic factor in T cell acute lymphoblastic leukaemia, nonsmall‐cell lung cancer (NSCLC), and other tumours [10, 11]. The elimination of this VAV1‐regulated tumour suppressor pathway is critical for the fitness of T cell acute lymphoblastic leukaemia of the TLX^+^ clinical subtype [12].

The catalytic and NFAT activities of VAV1 are activated by the phosphorylation by upstream tyrosine kinases of specific regulatory tyrosine residues located in its acidic (Ac), zinc finger (ZF), and CSH3 domains [1, 3, 13]. This process leads to a switch from a closed to an open conformation of the molecule that is now capable of interacting with the downstream GTPases and effectors [1, 3, 13, 14]. Due to this, mutations that eliminate the intramolecular interactions that maintain VAV1 in the close conformation result in the spurious activation of VAV1‐dependent signalling [13, 15, 16].

VAV1 has been historically linked to tumourigenesis due to its oncogenic activity in focus formation assays and, subsequently, from loss‐of‐function experiments that demonstrated the importance of the endogenous wild‐type protein in a number of cellular and mouse tumourigenic models [2, 15, 17, 18, 19, 20]. More recently, the sequencing of human tumours has revealed the presence at relatively high frequencies of *VAV1* mutations in peripheral T cell lymphoma (PTCL) subtypes such as angioimmunoblastic T cell lymphoma (AITL, 6% of total cases), adult T cell leukaemia/lymphoma (17% of total cases), and peripheral T cell lymphoma not otherwise specified (PTCL‐NOS, 7% of total cases). *VAV1* mutations have been also found at much lower frequency in other PTCL subtypes such as anaplastic large cell lymphoma (ALCL, 2% of total cases) and cutaneous T cell lymphoma (2% of total cases) [21, 22, 23, 24, 25, 26, 27]. In the case of lung tumours, *VAV1* mutations have been found at low frequencies in both lung adenocarcinomas (2.3% of cases) and squamous cell carcinomas (2.7% of cases) [28]. These *VAV1* mutations include missense changes, internal deletions due to splicing defects, C‐terminal truncations, and 3′‐end translocations with other genes. In a previous study, we have shown that most of those alterations lead to changes in the normal signalling of the protein [21, 29]. These mutations can be classified in three main subtypes depending on the impact on the downstream signalling of the protein: trivalent (with deregulated catalytic, NFAT, and tumour suppressor activities), bivalent (with deregulated catalytic and NFAT activities), and monovalent (which only show the inactivation of the suppressor pathway) [29]. Mutations of the trivalent and bivalent subclass are the most prevalent in human tumours [29]. Using adoptive T cell transfer experiments, we demonstrated that the expression of a trivalent subclass VAV1 mutant in CD4^+^ T cell can trigger *per se* the development of AITL *in vivo*. This protumourigenic effect requires the concurrent engagement of both the catalytic and the NFAT pathways by the oncogenic VAV1 mutants [29]. Using a transgenic mouse approach, Chiba's group also demonstrated that the ectopic expression of *Vav1* gain‐of‐function mutations favours T cell lymphomagenesis. However, in this case, this process requires the ablation of the *Trp53* tumour suppressor gene [30]. These observations raise a few additional questions: do VAV1 mutants promote tumourigenesis when expressed from the endogenous *Vav1* locus? If so, what is the spectrum of tumours that are driven by such mutations? Does this transformation require cooperating genetic lesions? Is this transformation the result of the generation of *ex novo* functions or the exacerbation of the normal functions played by wild‐type VAV1 in normal cells? To address those issues, we have generated by gene‐editing a mouse model that bears an oncogenic *Vav1* allele that encodes a trivalent subclass VAV1 mutant protein. Our results indicate that this mutant protein drives AITL formation and NSCLC progression. Furthermore, we show that the emergence of those tumours requires the cooperation of additional, tissue‐specific genetic lesions. Lastly, we demonstrate that the role of VAV1 in T cell lymphomagenesis is probably linked to the exacerbations of the physiological function of wild‐type VAV1 in T_FH_ cells.

## 2 Materials and methods

### 2.1 Ethics

All mouse experiments were performed according to protocols approved by the Bioethics Committee of the University of Salamanca (animal license number #568) and the animal experimentation authorities of the autonomous Government of Castilla y León (Spain). They were treated humanely in accordance with standards described in the Guide for the Care and Use of Laboratory Animals, considering relevant national and European guidelines. We have not utilized patients or patient‐derived samples in this work.

### 2.2 Generation of the *Vav1* ^ΔC^ mouse strain and other derivatives

To generate the *Vav1* ^ΔC^ mutant allele (Fig. S1A), we introduced by gene editing the sequence TAGTGACTAAGTGA downstream of the ACC codon present in the 27th exon of the *Vav1* gene that encodes the residue Asn^835^. To avoid stop codon skipping, we included two consecutive in‐frame stop codons followed by two additional ones in the other two reading frames (stop codons underlined) downstream of that codon. The *Vav1* synthetic guide RNA (sgRNA) targeting the sequence 5′‐CTG GTT CCC TTC TAA CTA TG‐3′ in *Vav1* exon 27 was generated by *in vitro* transcription from the *Vav1*‐gRNA plasmid. To generate this vector, we hybridized the oligonucleotides *Vav1*‐gRNA‐up (5′‐AGG GCT GGT TCC CTT CTA ACT ATG‐3′) and *Vav1*‐gRNA‐down (5′‐AAA CCA TAG TTA GAA GGG AAC CAG‐3′) to generate a double stranded DNA fragment with 5′ AGGG and 3′ TTTG overhangs to allow directional cloning into the BbsI sites of the gRNA basic plasmid [31]. Upon ligation, the insertion of the above sequences in the acceptor plasmid was confirmed by DNA sequencing. Subsequently, the *Vav1*‐gRNA was linearized with FspI and transcribed with T7 RNA polymerase using the MEGAshortscript™ kit (Cat. No. AM1354; Ambion, Austin, TX, USA) according to the manufacturer's protocol. The DNA template was then digested with DNaseI and the *Vav1*‐sgRNA purified with the MEGAclear™ RNA purification kit (Cat. No. AM1908; Ambion). The replacement single stranded DNA oligonucleotide (5′‐GAC AGT GGC TTT TCT GAT GGG TTT CTC TTC TCT CTG CAG ATC GGC TGG TTC CCT TCT AAC TAG TGA CTA AGT GAT ATG TGG AGG AAG ACT ATT CCG AAT ATT GCT GAG CCT GGT GCC CTG TAG GAC ACA GAG AGA GG‐3′), which contains the stop codons (underlined) flanked by genomic homology sequences consisting of the 60 nucleotides upstream and downstream of the 3′ end of the Asn^835^ codon, was obtained from IDT. The Cas9 protein was a gift from Juan Galcerán (Instituto de Neurociencias, CSIC, Alacant, Spain). To generate the mutant mice, a mix containing the *Vav1* sgRNA (10 ng·μL^−1^), the Cas9 protein (100 ng·μL^−1^) and the replacement oligo (10 ng·μL^−1^) in 10 mm Tris–HCl (pH 7.5) and 0.1 mm EDTA was introduced into fertilized C57BL/6 mouse oocytes by pronuclear microinjection. Identification of the recombinant allele in the resulting pups was done by PCR using genomic DNA purified from tail biopsies using the oligonucleotide pair *Vav1*‐Chk‐F (5′‐CAA CCT GCT GTG ATG AGG CAT G‐3′) and *Vav1*‐Rec‐R (5′‐CTC CAC ATA TCA CTT AGT CAC‐3′) that specifically detects the recombined allele. The *Vav1* genomic region was then reamplified by PCR using the diagnostic oligonucleotides *Vav1*‐Chk‐F and Vav1‐Chk‐R (5′‐CCT GGG CTC AGC CTT CAT CTG C‐3′) that can amplify the relevant *Vav1* genomic area from both the wild‐type (172 bp) and the mutant (186 bp) alleles. The PCR fragments were separated by electrophoresis in a 15% polyacrylamide/TBE gel and, after purification, the identity of the amplified band was further verified by DNA sequencing. The founder mouse was subsequently crossed with wild‐type C57BL/6 mice to generate the F_1_ generation bearing the *Vav1* mutant allele, which was again confirmed as above. Further crosses were performed to obtain a fully clean strain in the C57BL/6 genetic background. Genotyping was performed in all cases by PCR using the *Vav1*‐Chk‐F oligo with either the oligonucleotide *Vav1*‐Rec^WT^‐R (5′‐AGT CTT CCT CCA CAT AGT TAG‐3′) to identify the wild‐type allele or *Vav1*‐Rec‐R to identify the *Vav1* ^ΔC^ allele.

*Vav1* ^ΔC/ΔC^ mice were crossed with *Trp53* ^ER/ER^ [32] and LSL‐*Kras* ^G12D/+^ [33] mice to generate the compound *Trp53* ^ER/ER^;*Vav1* ^ΔC/ΔC^ and LSL‐*Kras* ^G12D/+^;*Vav1* ^ΔC/ΔC^ strains, respectively. *Vav1* ^−/−^ and *Vav1* ^−/−^;*Vav2* ^−/−^;*Vav3* ^−/−^ mice have been previously described [34, 35]. *Trp53* ^ER/ER^ and LSL‐*Kras* ^G12D/+^ mice were provided by D. Martín‐Zanca (Instituto de Biología Fundamental y Genómica, Salamanca, Spain) and M. Barbacid (Centro Nacional de Investigaciones Oncológicas, Madrid, Spain), respectively. Single *Vav1* ^−/−^ and *Vav2* ^−/−^ mice were gentle provided by V. Tybulewicz (Francis Crick Institute, London, UK) and M. Turner (Brabaham Institute, Cambridge, UK), respectively. *Vav3* ^−/−^ mice were generated in our own lab [36]. All mouse strains used were homogenized to the C57BL/6 genetic background, except *Vav1* ^−/−^ and *Vav1* ^−/−^;*Vav2* ^−/−^;*Vav3* ^−/−^ mice, which were both homogenized in the C57BL/10 genetic background. For all *in vivo* studies, female animals of the same genotype were randomly assigned to the different experimental groups. We did not exclude any animal from the final tabulation of results.

All mice were housed under specific pathogen‐free conditions with an artificial 12 h light/12 h dark cycle under controlled temperature (23 °C) and humidity (50%) conditions. They were routinely maintained under *ad libitum* access to a standard chow global diet (Teklad global 18% protein rodent diet; Cat. No. 2018; Envigo, Indiana, IA, USA) and tap water.

### 2.3 Isolation of primary mouse cells

These methods were described elsewhere [29]. Single cell suspensions were generated by mechanical homogenization of thymus, spleen, and lymph nodes in 3 mL of phosphate‐buffered saline solution supplemented with 2% bovine serum albumin and 0.5 mm EDTA (referred to hereafter as cell extraction buffer). Cells obtained were washed once by low‐speed centrifugation, resuspended in cell extraction buffer, and subjected to a 0.17 m NH_4_Cl lysis step to eliminate erythrocytes.

### 2.4 Western blot analyses

These experiments followed protocols described before [29]. Primary thymocytes and splenocytes were extensively washed with phosphate‐buffered saline solution and broken in lysis buffer 1 (10 mm Tris–HCl [pH 8.0], 150 mm NaCl, 1% Triton X‐100, 1 mm Na_3_VO_4_, 10 mm β‐glycerophosphate and a cocktail of protease inhibitors [Cømplete, Cat. No. 05056489001; Roche, Basel, Switzerland]). Cellular extracts were precleared by centrifugation at 20 000 *g* for 10 min at 4 °C, denatured by boiling in 2× SDS/PAGE sample buffer, separated electrophoretically, and transferred onto nitrocellulose filters (Cat. No. 2022‐04‐26; Thermo Fisher Scientific) using the iBlot Dry Blotting System (Thermo Fisher Scientific, Waltham, MA, USA). Membranes were blocked in 5% bovine serum albumin (Cat. No. A4503; Sigma‐Aldrich, Irvine, CA, USA) in TBS‐T (25 mm Tris–HCl [pH 8.0], 150 mm NaCl, 0.1% Tween‐20) for at least 1 h and then incubated overnight with the appropriate antibodies. Membranes were then washed three times with TBS‐T, incubated with the appropriate secondary antibody (GE Healthcare, Chicago, IL, USA) for 30 min at room temperature, and washed twice as above. Immunoreacting bands were visualized using a chemoluminescent method (ECL, Cat. No. RPN2209; Amershan, Chicago, IL, USA). Primary antibodies used included those to the VAV1 DH (homemade, 1 : 10 000 dilution), tubulin α (Cat. No. CP06‐100UG; Calbiochem, Darmstadt, Germany; 1 : 2000 dilution), TP53 (Cat. No. 2524; Cell Signaling Technologies, Danvers, MA, USA; 1 : 1000 dilution), PARP (Cat. No. 9542; Cell Signaling Technologies; 1 : 1000 dilution), and actin β (Cat. No. sc‐47778; Santa Cruz Biotechnology, Dallas, TX, USA; 1 : 1000 dilution).

### 2.5 Determination of mRNA abundance

Total RNA was extracted from cells using NZYol (Cat. No. MB18501; NZYtech, Lisbon, Portugal) and mRNAs purified using RNeasy Mini kit (Cat. No. 74106; Qiagen, Germantown, MA, USA) following the manufacturer's protocol. mRNAs were analysed by qRT‐PCR using the iScript One‐Step RT‐PCR kit with SYBR green (Cat. No. 1708892; BioRad, Hercules, CA, USA) and the StepOnePlus Real‐Time PCR System (Applied BioSystems, Waltham, MA, USA). Raw qRT‐PCR data were analysed using the stepone software v2.1 (Applied Biosystems), using the abundance of the endogenous *Actb* as internal normalization control. Primers used were: 5′‐TTG CCC AGA ACA AAG GAT C‐3′ (forward, *Vav1*), 5′‐AAG CGC ATT AGG TCC TCG TA‐3′ (reverse, *Vav1*), 5′‐GGC CAG CTG ATA TAA TGG AGA AAA‐3′ (forward, *Hes1*), 5′‐TCC ATG ATA GGC TTT GAT GAC TT‐3′ (reverse, *Hes1*), 5′‐CAC TGG CCC TGT CCA CCC AGC CTT GGC AGG‐3′ (forward, *Dtx1*), 5′‐ATG CGA ATT CGG GAA GGC GGG CAA CTC AGG‐3′ (reverse, *Dtx1*), 5′‐TCA GGT GTC AGG CTC TAC CA‐3′ (forward, *Ptcra*), 5′‐GTC CCT CAC CCT CCC AAA AG‐3′ (forward, *Actb*) and 5′‐GCT GCC TCA ACA CCT CAA CCC‐3′ (reverse, *Actb*).

### 2.6 Cycloheximide treatment

Single cell solutions from thymus and spleen were resuspended in RPMI‐1640 medium supplemented with 10% fetal bovine serum, 1% l‐glutamine, penicillin (10 μg·mL^−1^) plus streptomycin (100 μg·mL^−1^), and cultured in the presence of cycloheximide (Cat. No. C7698‐5G; Sigma‐Aldrich; 50 μg·mL^−1^) or solution control (DMSO). At the times indicated, cells were collected for western blot determinations as indicated above.

### 2.7 Flow cytometry determinations of surface and intracellular proteins

These techniques were previously described [12, 29]. Briefly, isolated cells were washed once in cell extraction buffer, resuspended in standard phosphate‐buffered saline solution, and stained following standard procedures with combinations of fluorescein isothiocyanate‐ (FITC, Cat. No. 553729; eBioscience, Waltham, MA, USA), allophycocyanin‐ (APC, Cat. No. 553051; eBioscience), APC‐Cy7‐ (Cat. No. 560181; eBioscience) or V500‐labelled (Cat. No. 560783; eBioscience) antibodies to CD4; FITC‐ (Cat. No. 553031; eBioscience), Pacific blue‐ (PB, Cat. No. 558106; eBioscience) or phycoerythrin‐labelled (PE, Cat. No. 553032; eBioscience) antibodies to CD8; APC‐ (Cat. No. 558643; eBioscience) or PE‐Cy7‐labelled (Cat. No. 552880; eBioscience) antibodies to CD25; perinidin chlorophyll‐cyanin 5.5‐labelled (PerCP‐Cy5.5) antibody to B220 (Cat. No. 45‐0452‐82; eBioscience); PE‐labelled antibody to TCRβ (Cat. No. 12‐5961‐82; eBioscience); FITC‐labelled antibody to GR1 (Cat. No. 11‐5931‐82; eBioscience); eFluor660‐labelled antibody to F4/80 (Cat. No. 50‐4801‐82; eBioscience); PE‐labelled antibody to PD1 (Cat. No. 12‐9985‐82; eBioscience); PE‐Cy7‐labelled antibody to CXCR5 (Cat. No. 25‐9185‐42; eBioscience); APC‐labelled antibody to ICOS (Cat. No. 17–9949‐82; eBioscience); V450‐labelled antibody to CD69 (Cat. No. 560690); FITC‐labelled antibody to GL7 (Cat. No. 144603; BioLegend, San Diego, CA, USA) and APC‐labelled antibody to CD95 (Cat. No. 17‐0951‐82; eBioscience).

For flow cytometry detection of phosphorylated intracellular proteins, cells were fixed with 2% formaldehyde, permeabilized with 90% ice‐cold methanol, and incubated with antibodies to p‐ERK1/2 (Cat. No. 4377; Cell Signaling Technologies; 1 : 200 dilution) and p‐AKT (p‐Ser^473^) (Cat. No. 4060; Cell Signaling Technologies; 1 : 400 dilution). Cells were then stained with an Alexa‐648‐labelled secondary antibody to rabbit immunoglobulins (Cat. No. A21443; Invitrogen, Waltham, MA, USA; 1 : 500 dilution). For intracellular ICN1 staining, cells were fixed with Cytofix/Cytoperm (Cat. No. 554714; BD Bioscience, Franklin Lakes, NJ, USA) for 10 min and stained with PE‐labelled antibodies to either TOX (Cat. No. 12‐6502‐82; eBiosciences; 1 : 50 dilution) or ICN1 (mN1A, Cat. No. 552768; Cell Signaling Technologies; 1 : 50 dilution) for 1 h at room temperature in phosphate‐buffered saline solution supplemented with 5% fetal bovine serum and 10% saponin. Unless otherwise stated, the antibodies used were from BD Biosciences. Antibody‐stained cells were run in a FACSAria III flow cytometer (BD Biosciences) and data analysed using the flowjo software (BD Biosciences).

### 2.8 Histological analyses

Mouse tissues were dissected and fixed in 4% paraformaldehyde in phosphate‐buffered saline solution, paraffin‐embedded, cut in 2–3 μm sections and subjected to haematoxylin–eosin staining using standard procedures. Sections were blindly analysed by an independent pathologist.

### 2.9 Treatment of animals with 4‐hydroxy‐tamoxifen

To restore TP53 expression in *Trp53* ^ER/ER^ and *Trp53* ^ER/ER^;*Vav1* ^ΔC/ΔC^ mice, we administered 1 mg of 4‐hydroxy‐tamoxifen dissolved in oil carrier daily by intraperitoneal injection for 1 week. Control mice were given oil carrier alone. Twenty‐four hours after the last administration, cells from spleen and lymph nodes were collected for flow cytometry determinations as indicated above.

### 2.10 Gene expression profiling

These experiments included splenic samples from control and lymphoma‐bearing animals. To this end, total RNAs from the spleen of *Trp53* ^ER/ER^ and *Trp53* ^ER/ER^;*Vav1* ^ΔC/ΔC^ mice were isolated using the RNAeasy Mini Kit (Catalog No. 74104; Qiagen) to be analysed using the Affymetrix platform (Clariom™ S Assay HT) at the CIC Genomics Core Facility according to the manufacture's recommendations.

### 2.11 Bioinformatics of microarray data

These procedures were described elsewhere [29]. r version 3.6.3 (R Core Team, www.r‐project.org) was used for statistical analyses along with python version 3.9 (The Python Foundation, www.python.org) for text file processing. Signal intensity values were obtained from expression microarray CEL files after robust multichip average. Differentially expressed genes were identified using linear models for microarray data (limma). Adjusted *P*‐values for multiple comparisons were calculated applying the Benjamini–Hochberg correction (FDR). Gene Ontology and KEGG pathways enrichment analyses were performed using DAVID (https://david.ncifcrf.gov). Expression heatmaps were generated using the heatmap3 R package. Volcano plots were generated using the Glimma R package. Gene set enrichment analysis (GSEA) were performed with described gene sets using gene set permutations (*n* = 1000) for the assessment of significance and signal‐to‐noise metric for ranking genes. The dataset used was the *Vav1* ^ΔC^‐associated gene signature generated in the adoptive transfer experiments with VAV1^ΔC^‐expressing CD4^+^ T cells [29]. To evaluate the gene signature fitness across the mouse *Rhoa* ^G17V^ and *Tet2* mutant CD4^+^ T cell signatures [37], the mouse GAPDH‐overexpressing T cell signature [38], the mouse FYN‐TRAF3IP2‐overexpressing bone marrow progenitors signature [39] and the mouse T_FH_ and T_H1_ cell signatures in the presence or absence of Tbx21 [40], the enrichment scores for both the upregulated and downregulated signatures found in *Trp53* ^ER/ER^;*Vav1* ^ΔC/ΔC^‐transformed AITL cells were calculated using single‐sample GSEA. The difference between the two normalized enrichment scores (NES) yielded the fit score, a measure of the enrichment and depletion of the upregulated and downregulated signatures, respectively. The *Trp53* ^ER/ER^;*Vav1* ^ΔC/ΔC^‐specific gene signature was established by comparing the full *Trp53* ^ER/ER^;*Vav1* ^ΔC/ΔC^‐associated gene signature with the differential transcriptomes of the signatures referred before.

### 2.12 CD4 ^+^ T cell isolation and analysis of normal T_FH_ cell responses

These methods were already described in a previous publication [29]. Single cell suspensions of spleen and lymph nodes from wild‐type, *Vav1* ^−/−^ and *Vav1* ^−/−^;*Vav2* ^−/−^;*Vav3* ^−/−^ mice were generated as above. Naïve CD4^+^ T cells were then purified by negative selection using the EasySep™ Mouse CD4^+^ T Cell Isolation Kit (Cat. No. 19852; StemCell Technologies, Vancouver, BC, Canada) according to the manufacturer's protocol. 1 × 10^6^ of purified CD4^+^ T cells were then stained with Cell Trace Violet (Cat. No. C34557; Life Technologies, Waltham, MA, USA) following the manufacturer's protocol and cultured on goat‐anti‐hamster IgG (Cat. No. 31115; Invitrogen)‐coated plate for 16–24 h in RPMI‐1640 media containing 10% fetal bovine serum, glutamine, 50 μm 2‐mercaptoethanol, antibodies to CD3 (Cat. No. 100202; BioLegend; 1 μg·mL^−1^), and antibodies to CD28 (Cat. No. 102102; BioLegend; 0.5 μg·mL^−1^). The quality of the purification step was always confirmed by flow cytometry. Upon activation, cells were washed and cultured in the presence of interleukin 2 (Cat. No. 200‐02; PeproTech, Rocky Hill, NJ, USA; 50 U·mL^−1^) and, 4 days later, were processed to measure the indicated biological and signalling parameters by flow cytometry.

### 2.13 Measurement of germinal centre response

NP‐keyhole limpet haemocyanin (Cat. No. N‐5060; Biosearch Technologies, San Francisco, CA, USA; 2 mg·mL^−1^) was mixed well with Complete Freund's adjuvant (Cat. No. F5881; Sigma‐Aldrich; 2 mg·mL^−1^) in a ratio 1 : 1 and administrated subcutaneously in the base of the tail of wild‐type, *Vav1* ^−/−^ and *Vav1* ^−/−^;*Vav2* ^−/−^;*Vav3* ^−/−^ mice. Twelve days after immunization, single cell suspensions from lymph nodes were generated and germinal centre response was evaluated.

### 2.14 Development of *Kras*‐oncogene driven NSCLC

Intranasal inhalation of adenoviral vectors was performed using infections with adenoviral particles encoding the Cre recombinase (Ad5‐CMV‐Cre) in 9‐ to 12‐month‐old mice of the indicated genotypes. Adenoviral stocks were generated at the University of Iowa Viral Vector Core Facility (Iowa City, IA, USA). To this end, mice anesthetized with a single intraperitoneal injection of ketamine (100 mg·kg^−1^ of body weight) and xylazine (3 mg·kg^−1^ of body weight) were intranasally infected with 50 μL of the adenovirus preparation (4 × 10^7^ plaque‐forming units). Mice were euthanized at the indicated periods (9, 15, and 25 weeks after the adenoviral infection) or at the ethical endpoints. Lung sections were analysed blindly by a pathologist to determine the number and type of hyperplasia and NSLCs that were obtained in each experimental cohort.

### 2.15 Immunohistochemistry and immunofluorescence

For immunohistochemistry determinations, sections were dewaxed, microwaved in citrate buffer (pH 6.0), and incubated overnight with antibodies to CC10 (Cat. No. 07‐623; Sigma‐Aldrich; 1 : 1000 dilution), surfactant protein C (SPC) (Cat. No. ab90716; Abcam, Cambridge, MA, USA; 1 : 500 dilution), phospho‐Histone 3 (p‐Ser10) (Cat. No. 06‐570; Merck, Darmstadt, Germany; 1 : 100 dilution) or p‐ERK1/2 (Thr202/Tyr204) (Cat. No. 4376; Cell Signaling Technologies; 1 : 500 dilution) at 4 °C. Sections were then incubated with biotin‐conjugated secondary antibodies followed by the addition of the Vectastain Elite ABC reagent (Cat. No. PK‐6100; Vector Laboratories, Burlingame, CA, USA). The reaction product was visualized by incubating the sections in 0.025% 3.3′‐diaminobenzidine and 0.003% H_2_O_2_ in phosphate‐buffered saline solution. For immunofluorescence determinations, sections were treated as described above. After incubation of the primary antibody to ICN1 (D3B8, Cat. No. 4147; Cell Signaling Technologies; 1 : 50 dilution), the fluorochrome‐conjugated secondary antibody (Cat. No. A‐21206; Thermo Fisher Scientific; 1 : 100 dilution) was applied for 1 h at room temperature. The percentage of p‐histone 3 and ICN1‐positive cells was quantified in the same area corresponding to four different microscopy fields per lung using the imagej software (NIH, Bethesda, MD, USA; www.imagej.net).

### 2.16 Statistics

Calculations were performed using Microsoft excel 2020 (Microsoft, Redmond, WA, USA) and graphpad prism software (version 6.0; Dotmatics, Boston, MA, USA) as previously described [29]. The number of biological replicates (*n*), the type of statistical tests performed, and the statistical significance are indicated in the figure legends. Parametric and nonparametric distributions were analysed using Student's *t*‐test and Mann–Whitney test, respectively. Chi‐squared tests were used to determine the significance of the differences between expected and observed frequencies. The Tukey's honest significance difference test was used to identify groups showing differential enrichment of the indicated signatures. Statistical analyses of the immunoblot‐generated data were carried out using the graphpad prism software (version 6.0). In all cases, values were considered significant when *P* ≤ 0.05. Data obtained are given as the mean ± SEM.

## 3 Results

### 3.1 Generation of VAV1^ΔC^ ‐expressing gene‐edited mice

To assess the spectrum of tumours induced by the expression of VAV1 gain‐of‐function mutations in cancer development, we used the CRISPR‐Cas9 gene editing technique (Fig. S1A) to generate mice bearing a *Vav1* mutant allele encoding a CSH3‐truncated protein (amino acids 835–845, referred to hereafter as VAV1^ΔC^) (Fig. S1B). This mutant protein shows high levels of both RAC1 and NFAT activity whereas it lacks the ability to suppress ICN1 signalling [13], thus behaving as a classical *VAV1* mutation of the so called trivalent functional subclass. This subclass is the most frequently found in AITL, PTCL‐NOS, ALCL, and lung tumours [29]. We have shown before that the expression of VAV1^ΔC^ in adult CD4^+^ T cells can drive *per se* the development of an angioimmunoblastic lymphoma (AITL)‐like condition in mice [29], further demonstrating the pathogenic nature of this functional subtype of VAV1 mutant proteins.

We found using immunoblot analyses that VAV1^ΔC^ is expressed at lower levels than the wild‐type counterpart when expressed in mice (Fig. S1C), a feature that is typically found in most VAV1 gain‐of‐function mutants when expressed in cells [29]. This differential expression seems to be caused by higher levels of instability of the VAV1 mutant version rather than to lower levels of its transcript (Fig. S1D,E). However, these reduced levels are sufficient to maintain the normal physiological functions of the protein as *Vav1* ^ΔC/ΔC^ animals exhibit the expected percentages of both immature (Fig. S2A) and mature (Fig. S2A–C) T cell subpopulations. They also contain normal levels of B lymphocytes (Fig. S2D,E), macrophages (Fig. S2F,G), and granulocytes (Fig. S2H,I). Thus, unlike the case of *Vav1* ^−/−^ mice [41], *Vav1* ^ΔC/ΔC^ animals do not develop T cell lymphopenia.

### 3.2 Expression of VAV1^ΔC^ promotes CD4 ^+^ T cell lymphomagenesis

**Fig. 1:**
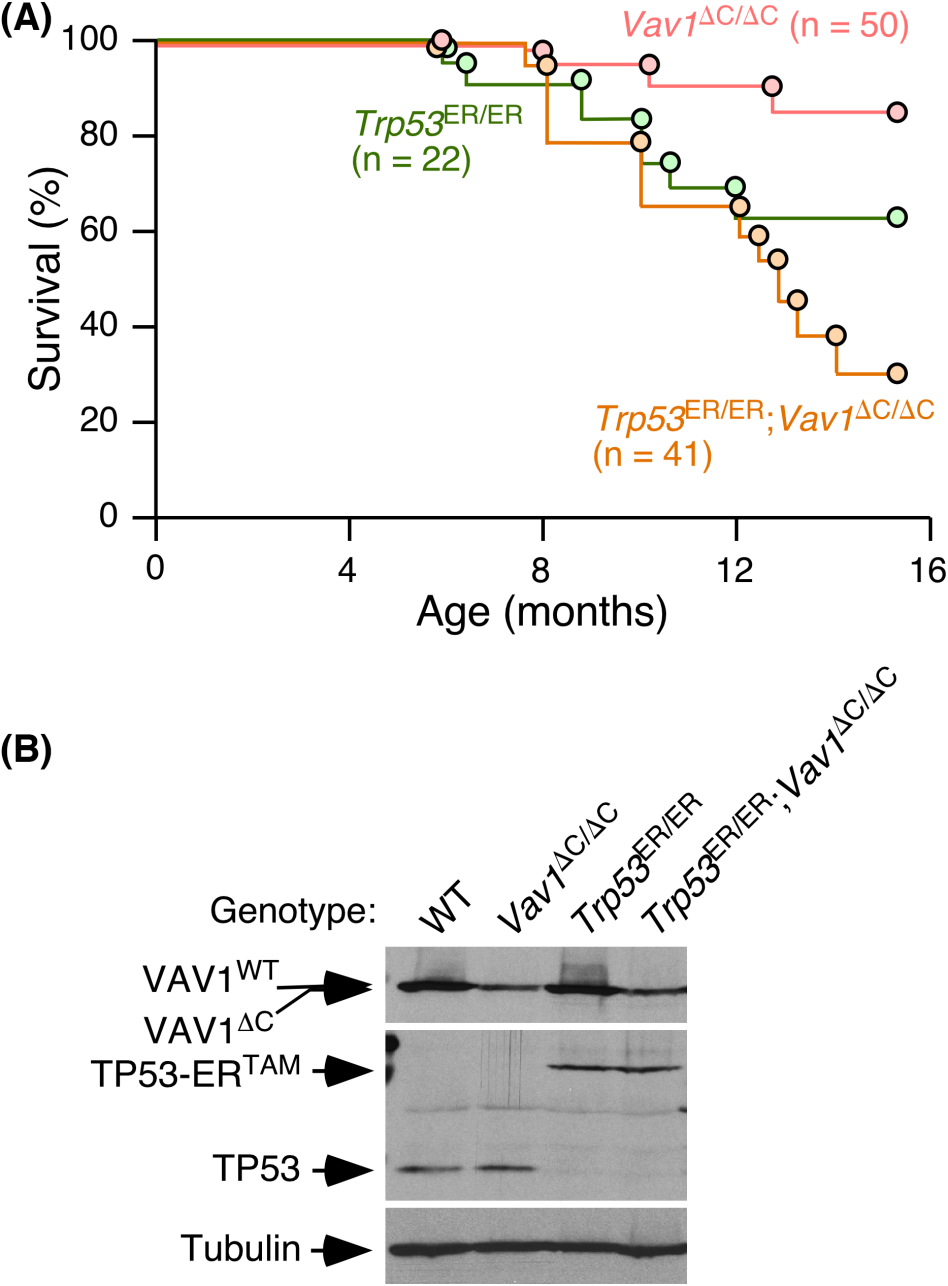
Low survival rates of *Trp53* ^ER/ER^;*Vav1* ^ΔC/ΔC^ mice. (A) Survival curves of mice of indicated genotypes. The number of animals analysed in each group is indicated in the graph. (B) Representative western blot showing abundance of VAV1 (top), TP53 (middle) and tubulin α (loading control, bottom) in total extracts from the spleen of indicated 2‐month‐old mice. Similar data were obtained in two additional independent experiments. [Colour figure can be viewed at wileyonlinelibrary.com]

**Fig. 2:**
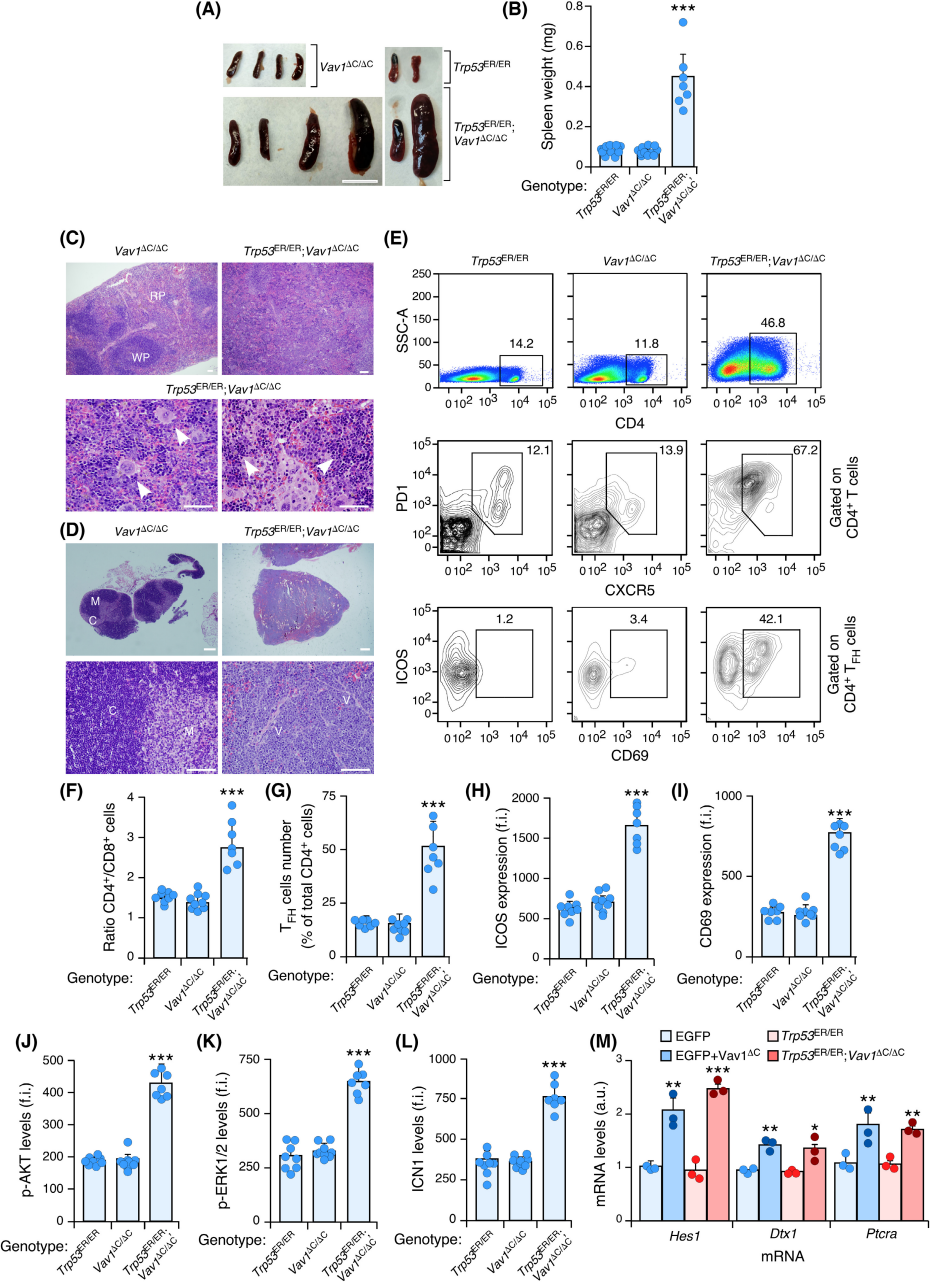
Expression of VAV1^ΔC^ promotes CD4^+^ T cell lymphomagenesis. (A, B) Representative view (A) and quantification of the weight (B) of spleen from mice of the indicated phenotypes at the time of euthanasia. In A, scale bar = 1 cm. In B, each dot represents a single experimental mouse. *n* = 12 animals per genotype, except in the case of *Trp53* ^ER/ER^ *;Vav1* ^ΔC/ΔC^ mice (*n* = 7). (C, D) Example of haematoxylin–eosin‐stained sections of the spleen (C) and lymph nodes (D) from animals of the indicated genotypes (top) at the time of euthanasia. In C, arrows indicate the presence of both megakaryocytes and leukemic cells. RP, red pulp; WP, white pulp. In D, C, cortex; M, medulla; V, venules. Scale bars, 10 (C, D, top panels) and 100 (C, D, bottom panels) μm. *n* = 5 independent tissue sections per genotype. (E) Representative flow cytometry plots showing the levels of surface expression of CD4 (top panels), PD1 and CXCR5 (middle panels) and ICOS plus CD69 (bottom panels) in splenocytes isolated from mice of indicated genotypes (top) at the time of euthanasia. *n* = 10 animals per genotype. In all cases, the numbers indicate the relative percentage (%) of the interrogated cell subpopulation in total (top raw of panels) or gated CD4^+^ T (middle raw of panels) and CD4^+^ T_FH_ (bottom raw of panels) cells. *n* = 8 (*Trp53* ^ER/ER^), 9 (*Vav1* ^ΔC/ΔC^) and 7 (*Trp53* ^ER/ER^ *;Vav1* ^ΔC/ΔC^) mice. (F) Flow cytometry determination of the CD4^+^ versus CD8^+^ T cell ratio in spleen from the mice of the indicated genotypes (bottom) at the time of euthanasia. Each point represents the values obtained with a single experimental mouse. *n* as in E. (G) Quantification of the percentage of T_FH_ cells in the population of CD4^+^‐gated splenocytes from mice of the indicated genotypes (bottom) at the time of euthanasia. Each point represents the values obtained with a single experimental mouse. *n* as in E. (H, I) Flow cytometry determination of the surface levels of ICOS (H) and CD69 (I) in CD4^+^ T_FH_‐gated splenocytes from animals of indicated genotypes (bottom) at the time of euthanasia. f.i., mean fluorescence intensity relative to the isotype‐matched control antibody. In both panels, each point represents the values obtained with a single experimental mouse. *n* as in E. (J–L) Flow cytometry determination of p‐AKT (J), p‐ERK1/2 (K) and ICN1 (L) levels in CD4^+^ T_FH_‐gated splenocytes from 8‐month‐old mice of the indicated genotypes at the time of euthanasia. *n* as in E. (M) qRT‐PCR analyses showing the expression of indicated ICN1 targets in lymphoma cell samples from the mouse models shown in the inset. *n* = 3. Data information: In panels B and F–M, the values represent the mean ± SEM. Statistical values are given relative to appropriate control animals. *P ≤ 0.05; **P ≤ 0.01; ***P ≤ 0.001 (Chi‐squared test). [Colour figure can be viewed at wileyonlinelibrary.com]

To investigate whether VAV1^ΔC^ could promote *in vivo* tumourigenesis and, if so, the spectrum of VAV1^ΔC^‐driven tumours, we monitored cohorts of both male and female *Vav1* ^ΔC/ΔC^ mice until they reached a maximum of 2 years of age. In contrast with the adoptive T cell transfer experiments recently reported [29], we could not detect the formation of AITL or any other haematopoietic or solid tumours in *Vav1* ^ΔC/ΔC^ mice during all the period analysed (J. R. Robles‐Valero and X. R. Bustelo, data not shown). As a result, these animals exhibit normal survival curves throughout the time‐period analysed (Fig. 1A). This indicates that the expression of VAV1^ΔC^ is not oncogenic *per se* at least in the absence of additional genetic lesions. Given that the loss of the *TP53* gene is a common event in NSCLC and some PTCL subtypes [28, 42, 43, 44], we decided to evaluate whether the loss of this tumour suppressor gene could synergize with the gain‐of‐function *Vav1* mutation in tumour formation. To this end, we crossed *Vav1* ^ΔC/ΔC^ mice with a knock‐in strain (referred hereafter as *Trp53* ^ER/ER^) that expresses a 4‐hydroxytamoxifen‐regulated TP53‐estrogen receptor fusion protein (TP53‐ER^TAM^) [32]. This protein is mostly inactive in cells due to improper migration to the nucleus in the absence of 4‐hydroxytamoxifen, thus mimicking a loss‐of‐function *Trp53* mutation [32]. However, an important feature of this model is that *Trp53* ^ER/ER^ mice develop tumours at much longer latencies than *Trp53* ^−/−^ mice [32] (Fig. 1A), thus allowing the visualization of synergistic interactions with mutations in other loci during longer time windows than when using standard *Trp53* ^−/−^ mice. Another important property of this model is that *Trp53* ^ER/ER^ mice do not develop mature T cell lymphomas [32], a feature that facilitates the analysis of the potential synergisms between *Vav1* gain‐of‐function and *Trp53* loss‐of‐function mutations in mature T cell lymphomagenesis without being affected by the background tumour noise usually caused when using *Trp53* ^−/−^ mice. Using this approach, we found that the compound *Trp53* ^ER/ER^;*Vav1* ^ΔC/ΔC^ mice exhibit lower survival rates than the *Vav1* ^ΔC/ΔC^ and *Trp53* ^ER/ER^ controls during the period analysed (Fig. 1A). This is not due to differential expression levels of either VAV1^ΔC^ (Fig. 1B, upper panel) or to the TP53‐ER fusion protein (Fig. 1B, second panel from top). The most prominent feature of the euthanized *Trp53* ^ER/ER^;*Vav1* ^ΔC/ΔC^ mice is the detection of splenomegaly and highly enlarged lymph nodes (Fig. 2A,B). Further histological analyses indicated that the spleen of these animals display in all cases effaced boundaries between the red and white pulp (Fig. 2C, top) as well as the frequent presence of both infiltrating lymphocytes and megakaryocytes (Fig. 2C, bottom). The lymph nodes also exhibit the effacement of cortico‐medulla boundaries and exacerbated levels of vascularization (Fig. 2D). We also found using flow cytometry determinations that *Trp53* ^ER/ER^;*Vav1* ^ΔC/ΔC^ mice exhibit abnormally high numbers of CD4^+^ T cells (Fig. 2E,F) that exhibit combinations of characteristic surface markers present in T_FH_ cells such as PD1 and CXCR5 (Fig. 2E,G). These lymphocytes are also highly decorated with ICOS (Fig. 2E,H) and CD69 (Fig. 2E,I), two surface markers typically associated with activated T_FH_ states. These histological and immunophenotypical features are consistent with the development of an AITL‐like condition in *Trp53* ^ER/ER^;*Vav1* ^ΔC/ΔC^ mice. Further flow cytometry determinations indicated that the transformed T_FH_ cells present in those mice show upregulated levels of phosphorylated (p‐) AKT (Fig. 2J), p‐ERK (Fig. 2K) and ICN1 (Fig. 2L) when compared with control *Trp53* ^ER/ER^ and *Vav1* ^ΔC/ΔC^ mice. All these histological, surface maker, and signalling features have also been observed in the AITL cells that are generated in adoptive transfer experiments with VAV1^ΔC^‐expressing CD4^+^ T cells [29]. Consistent with this, and as an example, we observed that the levels of expression of the canonical ICN1 target genes *Hes1*, *Dtx1*, and *Ptcra* are quite similar in the lymphoma cells from both mouse models (Fig. 2M). Collectively, these results indicate that the VAV1 trivalent gain‐of‐function and *Trp53* loss‐of‐function mutations cooperate in T cell lymphomagenesis *in vivo*.

### 3.3 Molecular features of AITLs arising in *Trp53* ^ER^ ^/ER^; *Vav1* ^ΔC^ ^/ΔC^ mice

**Fig. 3:**
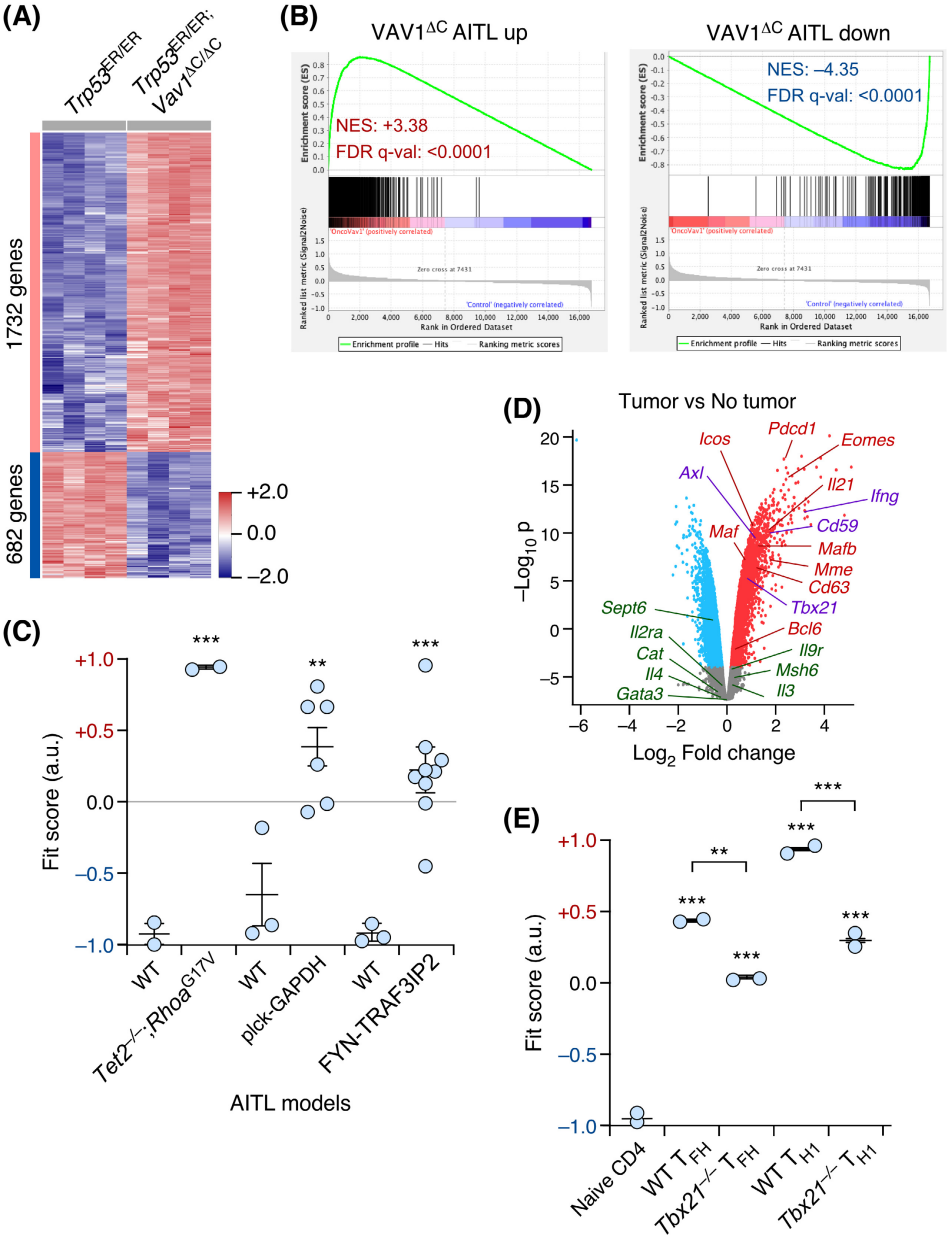
Molecular features of the AITL arising in *Trp53* ^ER/ER^;*Vav1* ^ΔC/ΔC^ mice. (A) Heatmap showing the genes that are up‐ (red) and downregulated (blue) in the tumoural cells obtained from the spleen of *Trp53* ^ER/ER^;*Vav1* ^ΔC/ΔC^ mice. As control, we used cells isolated from the spleen of *Trp53* ^ER/ER^ animals. Rows represent individual genes. Triplicates for each cell line (columns) were utilized. Total number of genes is indicated on the left. Relative changes in abundance are shown in colour gradients according to the colour scale located on the right. (B) GSEA showing the high overlap of indicated gene signatures in the transcriptomes of AITL cells obtained from *Trp53* ^ER/ER^;*Vav1* ^ΔC/ΔC^ mice and the previously described adoptive T cell transfer experiments [29]. The NES and false discovery rate values (FDR, using *q* values) are indicated inside the GSEA graph. Relative changes in abundance are shown in colour gradients according to the colour scale shown at the bottom. *q*‐val, *q* value. (C) Dot plot of the tumour‐specific *Trp53* ^ER/ER^;*Vav1* ^ΔC/ΔC^ gene signature fit score for indicated experimental groups (bottom). Dots represent values from an individual sample. Bars represent the mean enrichment value ± SEM for the overall sample set. **P ≤ 0.01; ***P ≤ 0.001 (Tukey's honest significance difference test). (D) Volcano plot showing genes that are significantly differentially expressed in the tumour cells from spleen of *Trp53* ^ER/ER^;*Vav1* ^ΔC/ΔC^ mice when compared with control samples. Up‐ or downregulated genes are depicted in red or blue, respectively. The colour code used for the indicated genes in the plot is related to the tumour subtype of PTCL patients. Red colour, PTCL‐T_FH_; violet colour, TBX21^+^ PTCL‐NOS; green colour, GATA3^+^ PTCL‐NOS. (E) Dot plot of the *Trp53* ^ER/ER^;*Vav1* ^ΔC/ΔC^‐dependent gene signature fit score in the indicated experimental groups (bottom). Data are presented as in panel C. **P ≤ 0.01; ***P ≤ 0.001 (Tukey's honest significance difference test). [Colour figure can be viewed at wileyonlinelibrary.com]

Consistent with the immunophenotypic and signalling similarities between the AITL cells generated in *Trp53* ^ER/ER^;*Vav1* ^ΔC/ΔC^ mice and animals transplanted with VAV1^ΔC^‐transduced CD4^+^ T cells, we also found using genome‐wide expression profiling experiments that the transcriptomes of the AITL cells obtained in each of those conditions are highly similar (Fig. 3A,B). As expected [29], we also found using *in silico* analyses a high level of similarity of these transcriptomes with other AITL models such as those derived from RhoA^G17V^‐expressing *Tet2* ^−/−^ CD4^+^ T cells and mice overexpressing GAPDH in the T cell compartment [37, 38] (Fig. 3C). Much lower levels of similarity were found with a GATA3 subtype‐like PTCL‐NOS obtained from the expression of the FYN‐TRAF3IP2 fusion protein in bone marrow progenitors [45] (Fig. 3C). Consistent with the development of an AITL condition, the transcriptome of the lymphoma samples obtained from *Trp53* ^ER/ER^;*Vav1* ^ΔC/ΔC^ mice includes the upregulation of AITL‐specific genes such as *Bcl6*, *Maf*, *Il21*, *Pdcd1*, and *Icos* [46] (Fig. 3D). Interestingly, the upregulated transcriptome of these lymphomas also includes the transcriptional factor TBX21 (also known as T‐BET) (Fig. 3D), the cotranscriptional regulator EOMES (Fig. 3D), and TBX21‐dependent gene signatures [40] (Fig. 3E). TBX21 is a transcriptional factor that, depending on environmental cues and the type of virus that CD4^+^ lymphocytes react against, can play antagonistic roles in the generation of type I T_FH_ cells and T_H1_ cells [40, 47, 48]. The presence of these molecular markers, therefore, suggest that the AITL driven by VAV1^ΔC^ derives from the transformation of type I T_FH_ lymphocytes. By contrast, these tumours lack the typical markers for GATA3^+^ PTCL‐NOS such as *Il2ra*, *il9r*, and *Gata3* itself [42] (Fig. 3D). These AITL‐like tumours do not arise in the single *Trp53* ^ER/ER^ mice, whose death is mostly due to the development of intrathymic lymphomas, skin tumours, and uncharacterized causes (J. R. Robles‐Valero, A. Abad, and X. R. Bustelo, data not shown). Based on these analyses, we conclude that VAV1^ΔC^ drives a common AITL‐like condition irrespectively of whether it is ectopically expressed in CD4^+^ T cells or endogenously expressed in mice. However, in the latter case, the emergence of the AITL‐like condition requires the lack of proper levels of TP53 tumour suppression activities.

### 3.4 Ontology of AITL formation in *Trp53* ^ER^ ^/ER^; *Vav1* ^ΔC^ ^/ΔC^ mice

**Fig. 4:**
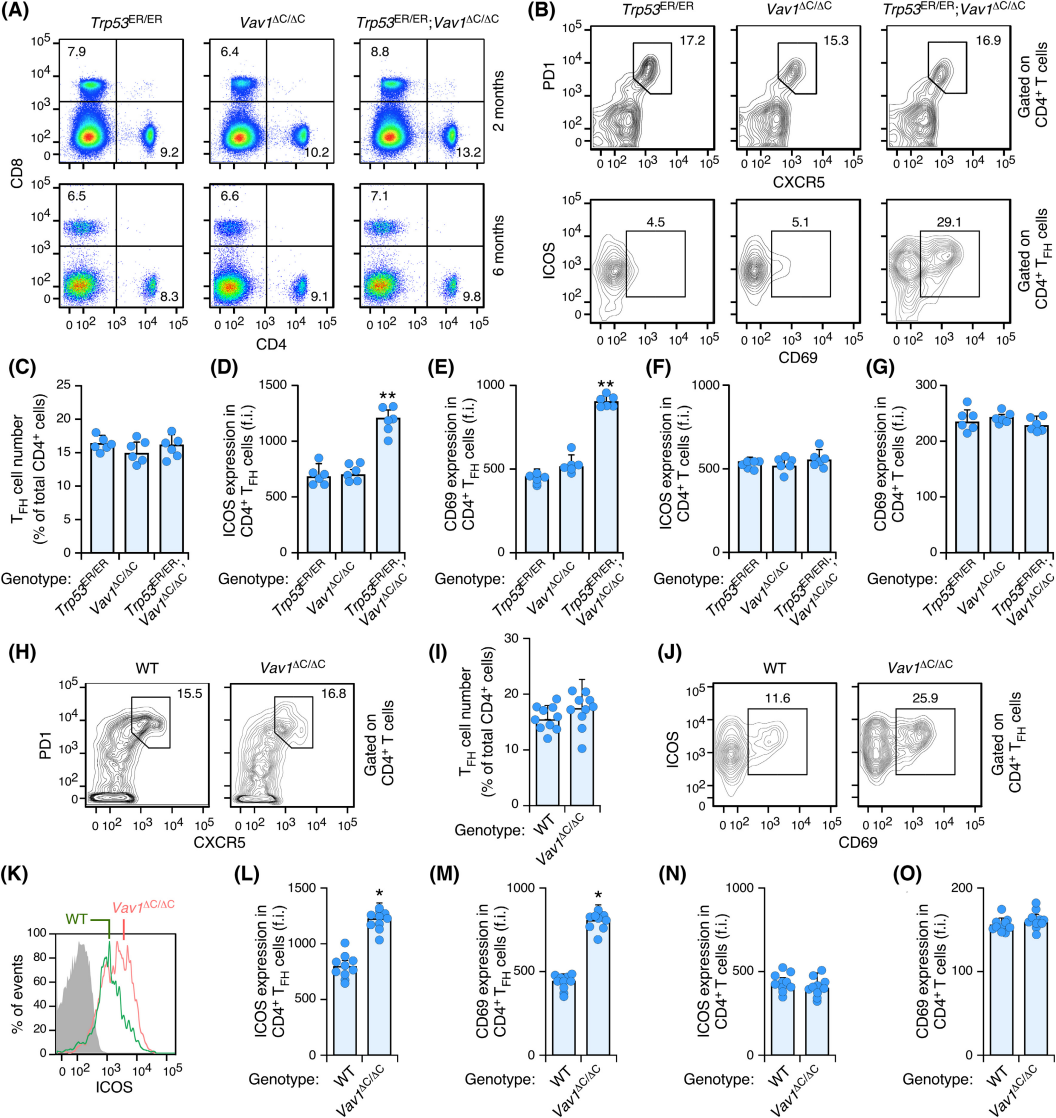
Ontology of AITL formation in *Trp53* ^ER/ER^;*Vav1* ^ΔC/ΔC^ mice. (A) Example of a flow cytometry analysis of the surface expression of CD8 and CD4 markers in peripheral blood cells from mice of the indicated genotypes. Animals were analysed at the ages indicated on the right. *n* = 6 mice per genotype analysed. (B) Representative FACS plots of surface expression of PD1 and CXCR5 (top panels) or ICOS and CD69 (bottom panels) in the gated populations (right) of splenocytes isolated from the 6‐month‐old mice of the indicated genotypes (*n* = 6 animals per genotype analysed). Numbers indicate the relative percentage (%) of the cell population selected. (C) Quantification of T_FH_ cell numbers (percentage) in CD4^+^‐gated splenocytes isolated from the 6‐month‐old mice of the indicated genotypes. Circles represent values from individual mice. *n* = 6 independent determinations/genotype. (D–G) Quantification of ICOS (D, F) and CD69 (E, G) abundance in CD4^+^ T_FH_ (D, E) and CD4^+^ T (F, G) splenocytes isolated from 6‐month‐old mice of the indicated genotypes. Circles represent values from individual mice. *n* = 6 independent determinations/genotype. (H, I) Example of the flow cytometry determination (H) and the quantification (I) of T_FH_ cells in CD4^+^‐gated splenocytes isolated from 12‐month‐old mice of the indicated genotypes. Circles represent values from individual mice. *n* = 10 independent determinations/genotype. (J–O) Example of a flow cytometry determination (J, K) and final quantification (L–O) of ICOS (J, K, L, N) and CD69 (J, M, O) surface expression in CD4^+^ T_FH_ (J–M) and CD4^+^ T (N, O) splenocytes isolated from 12‐month‐old mice of the indicated genotypes. Circles represent values from individual mice. f.i., mean fluorescence intensity relative to the isotype‐matched control antibody. *n* = 10 independent determinations/genotype. Data information: In panels C, D, E, F, G, I, L, M, N, and O, values represent the mean ± SEM. In panels C, D, E, F, and G, statistical values are given relative to the control *Trp53* ^ER/ER^ mice. In panels I, L, M, N, and O, statistical values are given relative to the control WT mice. *P ≤ 0.05; **P ≤ 0.01 (Chi‐squared test). [Colour figure can be viewed at wileyonlinelibrary.com]

The emergence of these AITL‐like tumours in *Trp53* ^ER/ER^;*Vav1* ^ΔC/ΔC^ mice requires long latency times, since we could not detect any significant alteration in the overall numbers of T cells (Fig. 4A) and PD1^+^ CXCR5^+^ T_FH_ populations (Fig. 4B,C) in 2‐ and 6‐month‐old *Trp53* ^ER/ER^;*Vav1* ^ΔC/ΔC^ mice. At this latter time, however, the T_FH_ cells are already in an activated ICOS^+^ (Fig. 4B,D) and CD69^+^ (Fig. 4B,E) state. Such an upregulation is not observed when using ungated populations of CD4^+^ lymphocytes (Fig. 4F,G). A similar population of PD1^+^ CXCR5^+^ ICOS^+^ CD69^+^ cells is found in 1‐year‐old *Vav1* ^ΔC/ΔC^ mice (Fig. 4H–O), suggesting that the faster development of tumours found in *Trp53* ^ER/ER^;*Vav1* ^ΔC/ΔC^ mice is probably due to the shortening in approximately 6 months of the time required for the expansion of the population of chronically activated T_FH_ cells found in 1‐year‐old *Vav1* ^ΔC/ΔC^ animals. Interestingly, the activated nature of the T_FH_ cells present in preneoplastic stages is not eliminated upon the tamoxifen‐dependent reexpression of TP53 in *Trp53* ^ER/ER^;*Vav1* ^ΔC/ΔC^ animals (Fig. S3). This suggests that the synergism observed between VAV1^ΔC^ and the loss function of TP53 does not take place at this early lymphomagenic phase.

### 3.5 VAV1^ΔC^ exacerbates normal functions of wild‐type VAV1 in T_FH_ cells

**Fig. 5:**
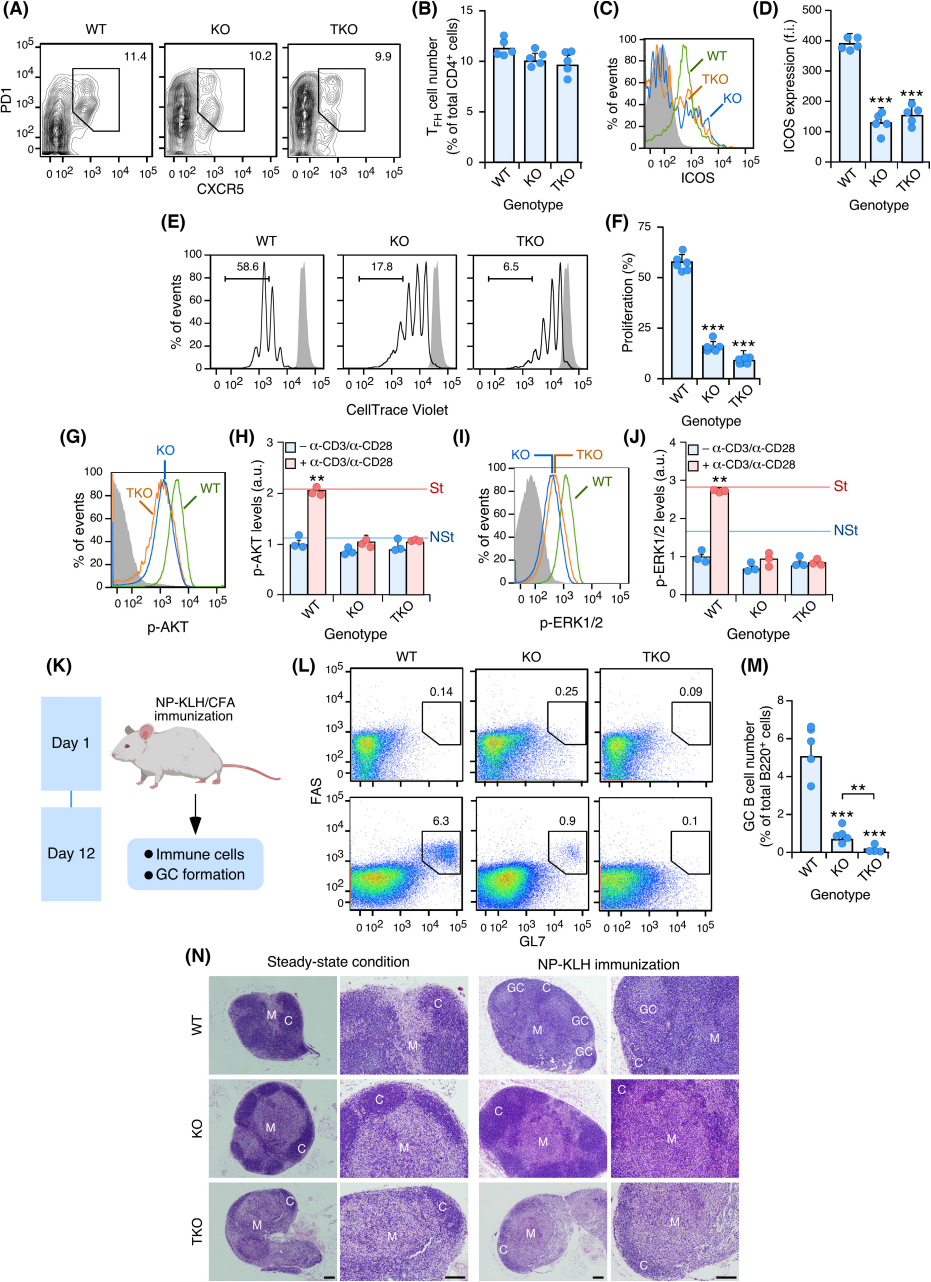
VAV1^ΔC^ exacerbates normal functions of wild‐type VAV1 in T_FH_ cells. (A) Flow cytometry analysis of expression of PD1 and CXCR5 in CD4^+^‐gated splenocytes isolated from 2‐month‐old mice of indicated genotypes (*n* = 5 animals per genotype). Numbers indicate the relative percentage (%) of the cell population selected. (B) Quantification of the percentage of T_FH_ cell numbers in CD4^+^‐gated splenocytes isolated from 2‐month‐old mice of the indicated mice. Each point represents the values obtained with a single experimental mouse. *n* = 5 animals per genotype. (C, D) Example of the flow cytometry detection (C) and quantification (D) of ICOS expression in T_FH_ cells isolated from 2‐month‐old mice of indicated genotypes. In D, each point represents the values obtained with a single experimental mouse. *n* = 5 animals per genotype. (E, F) Representative FACS plots (E) and quantification (F) of the proliferation of CD4^+^ T cell isolated from 2‐month‐old mice of indicated genotypes using the Cell Trace Violet method. Numbers indicate the relative percentage (%) of the CD4^+^ cell population in each case. Grey‐shaded histograms represent the fluorescence obtained from CD4^+^ T cells before activation. In F, each point represents the values obtained with a single experimental mouse. *n* = 5 animals per genotype. (G–J) Flow cytometry analysis (G, I) and quantification of p‐AKT (G, H) and p‐ERK1/2 (I, J) levels in CD4^+^ T_FH_ cells isolated from 2‐month‐old mice of indicated genotypes under both nonstimulation (blue bars) and TCR plus CD28‐stimulation conditions (red bars). NSt, nonstimulated; St, stimulated. *n* = 3 animals per genotype. (K) Depiction of immunization experiment used in this figure. Briefly, NP‐keyhole limpet haemocyanin (KLH) was administrated subcutaneously in the indicated genotypes and, 12 days after immunization, single cell suspensions and germinal centre response in lymph nodes was evaluated as indicated in Section 2. (L) Flow cytometry analysis of expression of FAS (CD95) and GL7 in B220^+^‐gated cells from lymph nodes isolated from 2‐month‐old mock‐injected (upper plots) and NP‐KLH‐injected (bottom plots) mice of the indicated genotypes. Numbers indicate the relative percentage (%) of the cell population selected. *n* = 5 animals per genotype, except in the case of *Vav1* ^−/−^;*Vav2* ^−/−^;*Vav3* ^−/−^ mice (*n* = 4). (M) Quantification of the percentage of germinal centre B cells in B220^+^‐gated cells isolated from lymph nodes isolated from 2‐month‐old mice of the indicated genotypes. Each point represents the measurement of an individual mouse. *n* as in L. (N) Representative examples of haematoxylin–eosin‐stained sections of lymph nodes isolated from 2‐month‐old mice mock‐injected (steady‐state condition) and NP‐KLH‐injected mice of the indicated genotypes (left) at the time of euthanasia. Scale bars, 100 μm. *n* = 3 animals per class analysed. C, cortex; M, medulla; GC, germinal centre. Data information: In panels B, D, F, and M, values are shown as mean ± SEM from three independent experiments. Statistical values obtained using the Student's *t* test are given relative to control group. In panels H and J, values are shown as mean ± SEM from three independent experiments and *P* values are given relative to nonstimulated and stimulated cells. We also included *P* values for the data obtained with each genotype relative to those obtained in nonstimulated conditions (black asterisks). **P ≤ 0.01; ***P ≤ 0.001 (Mann–Whitney *U* test). [Colour figure can be viewed at wileyonlinelibrary.com]

To distinguish whether the biological programs elicited by VAV1^ΔC^ during T_FH_ cell transformation were created *ex novo* or were the reflection of the exacerbation of the normal functions of wild‐type VAV1 in T cells, we analysed T_FH_ cell responses in both *Vav1* ^−/−^ and *Vav1* ^−/−^;*Vav2* ^−/−^;*Vav3* ^−/−^ mice. These two knock‐out strains exhibit ratios of T_FH_/total CD4^+^ T cells identical to those found in wild‐type mice (Fig. 5A,B). However, the T_FH_ cells from the two knockout mice display 4‐fold lower levels of ICOS expression than controls (Fig. 5C,D). They also show reduced levels of proliferation upon stimulation with TCR plus CD28 (Fig. 5E,F), a defect that goes in parallel with defective phosphorylation levels of both AKT (Fig. 5G,H) and ERK (Fig. 5I,J). VAV1 seems to be the family member more relevant in this process, since we have not observed statistically significant quantitative differences in the defects found in *Vav1* ^−/−^ and *Vav1* ^−/−^;*Vav2* ^−/−^;*Vav3* ^−/−^ mice in all those assays (Fig. 5A–J).

To further confirm these results using a scenario closer to normal physiological responses, we decided to assess the germinal centre response in animals of the indicated genotypes (Fig. 5K). To this end, mice were immunized by injecting NP‐keyhole limpet haemocyanin plus complete Freund's adjuvant and, 12 days later, the numbers of germinal centre B cells (B220^+^ FAS^+^ GL7^+^) were quantified by flow cytometry. We could not see any change in the numbers of germinal centre B cells in the mock‐injected mice regardless of the genotype involved (Fig. 5L, top panels). However, upon immunization, we observed that the generation of those cells was impaired in *Vav1* ^−/−^ mice and, to a larger extent, in the triple *Vav1* ^−/−^;*Vav2* ^−/−^;*Vav3* ^*−/−*^ knockout animals^.^(Fig. 5L, bottom panels; Fig. 5M). Consistent with this, we could not observe the formation of germinal centres in the lymph nodes of those two mouse strains upon the immunization protocol using histological analyses (Fig. 5N). Taken together, these results suggest that VAV1^ΔC^ promotes lymphomagenesis by exacerbating the activity of the signalling pathways that are regulated by wild‐type VAV1 under normal conditions in T_FH_ cells.

### 3.6 VAV1^ΔC^ favours progression of K‐RAS^G12D^ ‐driven lung tumours

**Fig. 6:**
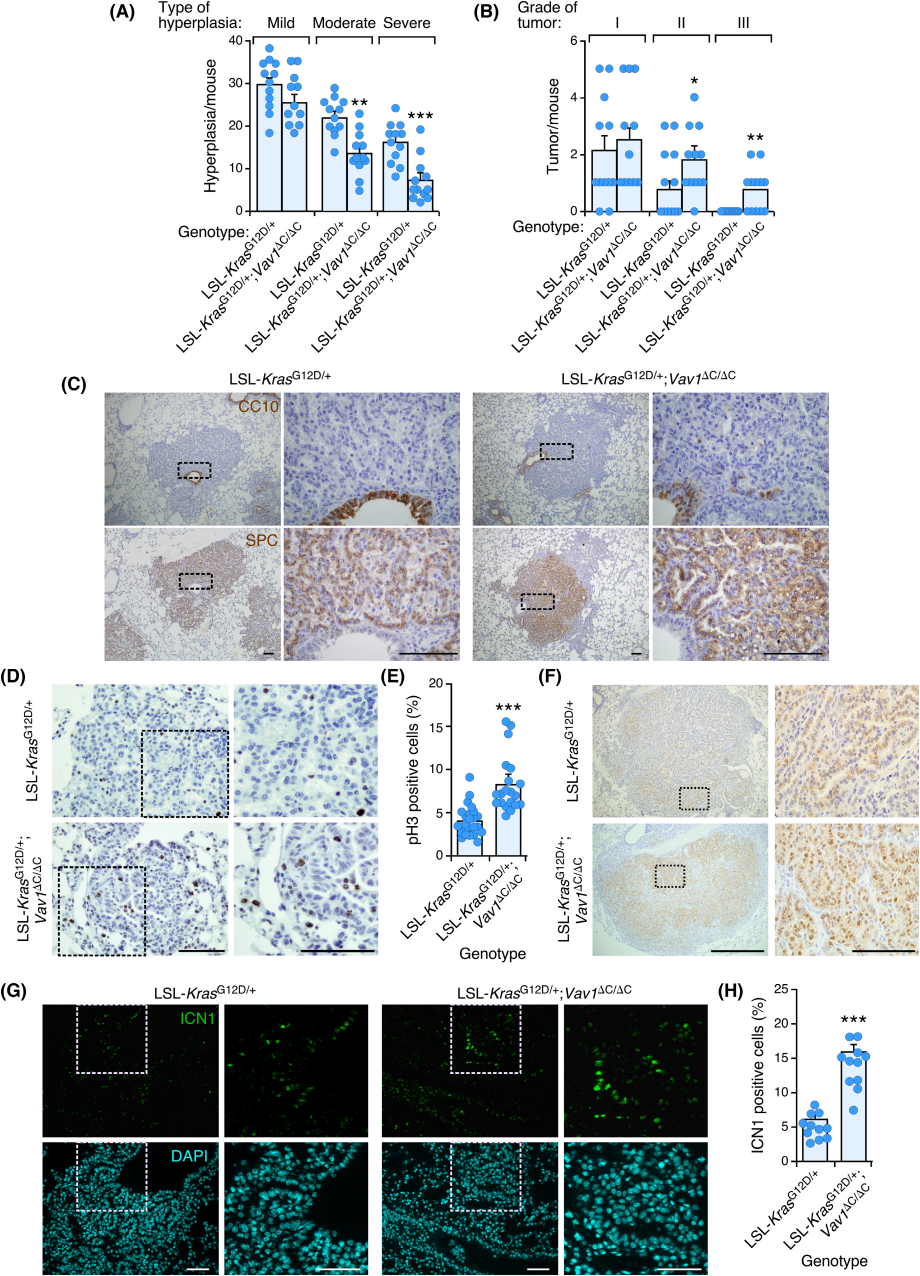
VAV1^ΔC^ favours progression of K‐RAS^G12D^‐driven lung tumours. (A, B) Quantification of type of hyperplasia (A) and grade of tumours (B) detected in the indicated genotypes at the time of euthanasia (25 weeks after the adenoviral infection). Each dot represents one mouse (*n* = 12 animals per genotype analysed). (C) Representative images of tumour‐harbouring lungs from the indicated genotypes at the time of euthanasia (25 weeks after the adenoviral infection) after staining with antibodies to CC10 (upper panels) and SPC (bottom panels). *n* = 3 independent tissue sections per genotype, 5 animals. Scale bars, 500 (left panels) and 100 (right panels) μm. (D, E) Example (D) and quantification (E) of the proliferation in tumours derived from the indicated genotypes at the time of euthanasia (25 weeks after the adenoviral infection) (*n* = 3 independent tissue sections per genotype, 5 animals). Sections were decorated with phospho‐H3 (pH3). Scale bars, 100 μm. (F) Representative images of tumour‐harbouring lungs from the indicated genotypes at the time of euthanasia (25 weeks after the adenoviral infection) after staining with an antibody to phospho‐ERK1/2 (*n* = 3 sections per tumour, 5 animals). Scale bars, 500 (left panels) and 100 (right panels) μm. (G, H) Example (G) and quantification (H) of the ICN1 immunostaining (upper panels, green colour) of lung tumours derived from the indicated genotypes at the time of euthanasia (25 weeks after the adenoviral infection) (*n* = 2 sections per tumour, 4 animals). In all cases, the nuclei of cells were labelled with DAPI (bottom panels, blue colour). Scale bars, 500 (left panels) and 100 (right panels) μm. Data information: In panels A, B, E, and H, values are shown as mean ± SEM from three independent experiments. Statistical values obtained using the Mann–Whitney *U* test are given relative to control mice (LSL‐*Kras* ^G12D/+^). *P ≤ 0.05; **P ≤ 0.01, ***P ≤ 0.001. [Colour figure can be viewed at wileyonlinelibrary.com]

Although *VAV1* mutations are found in NSCLC, we could not detect any lung tumours in *Vav1* ^ΔC/ΔC^ and *Trp53* ^ER/ER^;*Vav1* ^ΔC/ΔC^ mice. To further explore the involvement of VAV1^ΔC^ in these tumours, we crossed the *Vav1* ^ΔC/ΔC^ mice with a mouse strain that can express one of the oncogenic versions of K‐RAS (G12D) in a Cre recombinase dependent manner (referred hereafter as LSL‐*Kras* ^G12D/+^). It has been shown before that LSL‐*Kras* ^G12D/+^ mice develop NSCLC upon the intratracheal delivery of Cre‐encoding adenoviral particles [33, 49]. Using this experimental model, we found that the VAV1^ΔC^ promotes a faster progression of K‐RAS^G12D^‐driven NSCLCs. Consistent with this, LSL‐*Kras* ^G12D/+^;*Vav1* ^ΔC/ΔC^ mice display reduced levels of moderate and severe lung hyperplasia than controls 25 weeks after the adenoviral infection (Fig. 6A). Conversely, they exhibit higher frequencies of histological grade II and III NSCLCs than controls (Fig. 6B). The tumours developed in LSL‐*Kras* ^G12D/+^;*Vav1* ^ΔC/ΔC^ and control LSL‐*Kras* ^G12D/+^ mice are SPC^+^ and CC10^−^ in both cases (Fig. 6C). However, the tumours from LSL‐*Kras* ^G12D/+^;*Vav1* ^ΔC/ΔC^ mice are more proliferative (Fig. 6D,E) and contain higher levels of nuclear p‐ERK (Fig. 6E) and ICN1 (Fig. 6F,G) than those from single LSL‐*Kras* ^G12D/+^ animals. We did not find evidence of metastasis in lymph nodes or other tissues in any of these mice (M. Cuadrado and X. R. Bustelo, unpublished data). These results indicate that VAV1^ΔC^ can synergize with *Kras* oncogenic mutations in NSCLC progression.

## 4 Discussion

In this work, we confirmed using a gene‐edited mouse model that the trivalent functional subclass of *Vav1* mutations plays critical roles in the development of AITL. However, unlike the case of the adoptive T cell transfer experiments previously described [29], we have found that effective AITL formation in *Vav1* ^ΔC/ΔC^ mice requires the elimination of the suppressor activity of TP53. This VAV1^ΔC^‐driven lymphomagenic process involves long latency times, suggesting that the accumulation of further genetic alterations is required to generate full‐blown tumours in the *Trp53* ^ER/ER^;*Vav1* ^ΔC/ΔC^ mice. Additional take‐home messages can be derived from this work. One of them is that the oncogenic driver activity of the *Vav1* gain‐of‐function mutations is highly specific for specific tumours (AITL) and cell types (T_FH_ cells). The implication of VAV1 in the transformation of this cell lineage is probably linked to the important role that wild‐type VAV1 plays in both the polarization and physiological responses of T_FH_ cells. However, this selectivity is highly surprising given that VAV1 is normally expressed in most haematopoietic cells and plays roles in many T cell subtypes. The reason for this selectivity is unclear. It is possible that the VAV1‐mediated transformation of other cell lineages might require different subsets of cooperating genetic alterations. This possibility can apply for example to adult T cell leukaemia/lymphoma, whose development is heavily dependent on oncogenic events driven by the genomic integration of the human T‐cell leukaemia virus type‐1 [24]. However, the lack of tumour formation in other haematopoietic cell types might be the consequence of the rewiring of the developmental path of some cell lineages by oncogenic VAV1. Thus, it is possible that the induction of BCL6‐ and ICOS‐dependent programs by oncogenic VAV1 in CD4^+^ T cells could favour the polarization towards the T_FH_ lineage and, in turn, block the differentiation and transformation of T_H1_ and T_H2_ cells [50]. Less surprising is the lack of detection of solid tumours found in *Trp53* ^ER/ER^;*Vav1* ^ΔC/ΔC^ mice as the expression of wild‐type VAV1 is usually restricted to the haematopoietic compartment [51]. This suggest that *Vav1* oncogenic mutations will only affect the pathobiological properties of solid tumours that promote the spurious expression of this signalling protein. This is the case, for example, of NSCLC, neuroblastoma, and pancreatic cancer [18, 52, 53].

A second conclusion of our work is that the spectrum of tumours induced by VAV1^ΔC^ is highly reproducible regardless of the experimental approach used. Consistent with this, we have found an AITL‐like condition in *Trp53* ^ER/ER^;*Vav1* ^ΔC/ΔC^ mice that, according to all the anatomopathological, immunosurface, and transcriptomal features analysed, is undistinguishable to that previously found upon the transplantation of CD4^+^ T cells transduced with VAV1^ΔC^‐encoding retroviruses into recipient mice [29]. Importantly, these features are very similar to those found in mouse models that develop AITL and T_FH_ subtype PTCLs as well as in AITL patients. Despite this similarity, we have found that the generation of AITL when VAV1^ΔC^ is expressed from the endogenous locus requires defective *TP53* suppressor functions. A similar observation has been recently made by Chiba's group using transgenic mice [30]. The reason for these different genetic requirements in our two experimental models is unclear at present. One obvious explanation is that the ectopic expression of VAV1^ΔC^ used in the adoptive T cell transfer experiments can promote stronger signalling than the endogenous counterpart present in the gene‐editing model. However, if that were solely the case, it would be expected that the elimination of TP53 would be more beneficial in this case than in the gene‐edited mouse model. It is also possible that the elimination of TP53 function could allow bypassing negative signalling loops established during the embryonic period or the differentiation of T cells within the thymus to buffer the exacerbated signalling induced by the mutant protein. This problem would not exist in the case of the adoptive T cell transfer experiments, given that in this case VAV1^ΔC^ is expressed in mature cells that have already passed through all the intrathymic selection steps. In line with this, we have found that the reexpression of TP53 does not affect the activation state of T_FH_ cells in preneoplastic stages of *Trp53* ^ER/ER^;*Vav1* ^ΔC/ΔC^ mice. By contrast, it is unclear that this TP53‐dependency is associated to lower signalling levels of endogenous VAV1^ΔC^ as we found quite similar transcriptomal changes in the lymphoma cells obtained from *Trp53* ^ER/ER^;*Vav1* ^ΔC/ΔC^ mice and adoptively transferred VAV1^ΔC^‐expressing CD4^+^ cells. Likewise, similar levels of the expression of ICN1 target genes have been observed in both cases. Arguably, further studies will be needed to fully understand the contribution of the loss of TP53 to this process.

In contrast to our “clean” tumour formation pattern, the expression of VAV1 mutants using a transgenic approach leads to the formation of a much complex spectrum of immature and mature T cell lymphomas [30]. In addition, some of the mature lymphomas exhibit GATA3^+^ T_H2_ subtype molecular features although, in most cases, are negative for CD4 [30]. The mouse models used in these two works are, however, quite different. Thus, in our case, we have used mice in which the *Vav1* oncogenic mutation has been generated in the endogenous *Vav1* locus by gene‐editing. As a result, VAV1 is expressed in T cells following the same dynamics and timing that the wild‐type counterpart. By contrast, the transgenic mice express *Vav1* cDNAs under the regulation of the CD2 promoter. This might be relevant, since our mice do not exhibit the alterations in T cell development found in the transgenic mice [30]. Likewise, the different strategy used for eliminating the suppressor function of TP53 (expression of a defective TP53‐ER fusion protein rather than the total genetic inactivation of the *Trp53* gene) has enabled us to generate a model that does not have the high basal levels of tumour formation that are usually driven by the full *Trp53* knockout condition.

In addition to the above information, we believe that another important result of this work is the generation of the VAV1^ΔC^ mouse strain itself. This model will represent from now on an important tool to further study the etiology of AITL, a disease that is still poorly understood both by its molecular complexity and the rather limited number of mouse models that recapitulate the anatomopathological and molecular features of the tumours found in human tumours [54, 55]. In agreement with this idea, we have shown that the gene edited *Vav1* ^ΔC/ΔC^ mouse model gives further proof that AITL derives from the transformation of T_FH_ cells as previously proposed in other studies [29, 54, 56, 57, 58]. This cell lineage, which normally provides B cell costimulatory signals to favour the generation of optimal humoral responses to foreign antigens, develops from conventional CD4^+^ T lymphocytes after a complex differentiation process that entails migration steps between different lymph nodes areas and crosstalk with surrounding cells. The T_FH_ phenotype is highly plastic, exhibiting several states depending on their localization and interactions with a variety of cell types [59]. Our results have also unveiled that the CD4^+^ T cells present in *Trp53* ^ER/ER^;*Vav1* ^ΔC/ΔC^ mice progressively differentiate into T_FH_ cells that, with time, acquire a chronic activated state *in vivo*. These cells subsequently move into a phase of rapid expansion that causes the emergence of terminal disease and the death of the animals. In these late stages, the tumour bearing animals exhibit all the anatomopathological, cellular, and molecular features typically exhibited by human AITL. *Trp53* ^ER/ER^;*Vav1* ^ΔC/ΔC^ mice require long latency times to reach this terminal disease state, an observation that is also in syntony with the usual detection of this disease in old people [60]. It also suggests that, in addition to the *Vav1* and *Trp53* mutations, this lymphomagenic process likely requires the acquisition of further genetic lesions. Thus, the use of these mice can be interesting to achieve a good understanding of the cooperating mutations that drive the generation of a full‐blown disease. Likewise, this mouse model will be an excellent tool to further dissect mechanistic aspects of this disease as well as the testing of new anti‐AITL drugs. This mouse model will be also useful to study the influence of oncogenic VAV1 signalling on other tumour types and the type of cooperating mutations that are required to trigger tumour formation or progression.

It is likely that the implication of VAV1 on tumorigenic processes goes beyond the proactive roles played by its mutant versions. Thus, a variety of loss‐of‐function studies using cells or mouse models have demonstrated that the elimination of wild‐type VAV1 can impair the formation of specific tumor types [2, 17, 18, 19, 20, 53]. Conversely, the ectopic expression of wild‐type VAV1 via transgenic approaches has been shown to accelerate pancreatic and lung tumor formation when combined with oncogenic K‐RAS mutants [61, 62]. It is important to note, however, that these latter models are somewhat artificial given that they ectopically overexpress the protein using strong tissue‐specific promoters [61, 62]. As a result, the effects obtained might be different from those that are normally elicited by the endogenous protein under the same pathological conditions. On the other hand, it has been shown that wild‐type VAV1 can play tumor suppresor roles in other tumor types such as TCR‐negative T cell acute lymphoblastic leukemia [12]. Further work will be needed to fully understand the complex role of this key signaling protein in tumor formation and maintenance. In any case, all these data suggest that the inhibition of VAV1 could represent a potential therapeutic avenue to treat specific tumor types.

## 5 Conclusions

The data shown in this work demonstrate that *VAV1* gain‐of‐function mutations play driving roles in tumourigenesis. However, our results also indicate that such functions are highly cell and tumour type specific. Consistent with this, we have found that the expression of VAV1^ΔC^ from the endogenous locus only promotes the generation of AITL‐like tumours despite being expressed in multiple haematopoietic lineages throughout the embryonic and postnatal periods. Likewise, we have observed that the genetic determinants that cooperate in VAV1^ΔC^‐mediated tumourigenesis are tumour type specific. In agreement with this, we have shown that VAV1^ΔC^ requires different mutations to favour tumourigenesis in the T cell compartment (loss of *Trp53*) and in the lung (*Kras* oncogenic mutations). In the case of AITL, our data indicate that the role of VAV1^ΔC^ is probably linked to the exacerbation of the normal functions of wild‐type VAV1 in T_FH_ cell polarization and proliferation. By contrast, its role in NSCLC seems highly contingent on genetic or biological conditions that promote the spurious expression of the *Vav1* gene in the lung, a tissue that does not normally express this signalling protein.

Given the similarity of the VAV1^ΔC^‐driven AITL condition and human AITL subtype cases, our data also indicate that the animal model reported here can be highly valuable to further address the etiology of this disease and the identification of comutations that promote its final expansion phase. It will be also useful for the preclinical testing of drugs to use in both AITL and PTCL‐NOS (T_FH_ subtype) patients, two diseases that are in high need of new therapeutic options.

## Conflict of interest

The authors declare no conflict of interest.

## Author contributions

JR‐V participated in all experimental work, analysed data and contributed to both artwork design and manuscript writing. LF‐N, IF‐P, AA, and MD helped in animal model‐based experiments. MC, ER, LM, and AB characterized the NSCLC model. LFL‐M carried out the *in silico* analyses. DM‐Z and MM generated the *Trp53* ^ER/ER^ and the gene‐edited mice, respectively. XRB conceived the work, analysed data, wrote the manuscript, and carried out the final editing of figures.

6

### Peer Review

The peer review history for this article is available at https://publons.com/publon/10.1002/1878‐0261.13295.

## Supporting information


**Fig. S1.** Generation of the *Vav1* ^ΔC/ΔC^ mouse strain.
**Fig. S2.** *Vav1* ^ΔC/ΔC^ mice show normal lymphoid and myeloid populations.
**Fig. S3.** The activation state of TFH cells is maintained upon reexpression of TP53 in *Trp53* ^ER/ER^;*Vav1* ^ΔC/ΔC^ mice.

## Data Availability

A Source Data file for this article with all the raw data used for graphs and immunoblots is included in the online materials associated with this article. All relevant data are available from the corresponding author upon reasonable request. Microarray data reported in this article have been deposited in the GEO database (https://www.ncbi.nlm.nih.gov/geo/) under the accession number GSE189221.

## References

[1] Bustelo XR . Vav family exchange factors: an integrated regulatory and functional view. Small GTPases. 2014;5:9. 10.4161/21541248.2014.973757 25483299 PMC4601183

[2] Katzav S . Vav1: a Dr. Jekyll and Mr. Hyde protein – good for the hematopoietic system, bad for cancer. Oncotarget. 2015;6:28731–42. 10.18632/oncotarget.5086 26353933 PMC4745688

[3] Rodriguez‐Fdez S , Bustelo XR . The Vav GEF family: an evolutionary and functional perspective. Cells. 2019;8:465. 10.3390/cells8050465 PMC6562523 31100928

[4] Turner M , Billadeau DD . VAV proteins as signal integrators for multi‐subunit immune‐recognition receptors. Nat Rev Immunol. 2002;2:476–86. 10.1038/nri840 12094222

[5] Crespo P , Schuebel KE , Ostrom AA , Gutkind JS , Bustelo XR . Phosphotyrosine‐dependent activation of Rac‐1 GDP/GTP exchange by the vav proto‐oncogene product. Nature. 1997;385:169–72. 10.1038/385169a0 8990121

[6] Kuhne MR , Ku G , Weiss A . A guanine nucleotide exchange factor‐independent function of Vav1 in transcriptional activation. J Biol Chem. 2000;275:2185–90. 10.1074/jbc.275.3.2185 10636924

[7] Wu J , Katzav S , Weiss A . A functional T‐cell receptor signaling pathway is required for p95vav activity. Mol Cell Biol. 1995;15:4337–46. 10.1128/mcb.15.8.4337 7623828 PMC230673

[8] Martinez GJ , Hu JK , Pereira RM , Crampton JS , Togher S , Bild N , et al. Cutting edge: NFAT transcription factors promote the generation of follicular helper T cells in response to acute viral infection. J Immunol. 2016;196:2015–9. 10.4049/jimmunol.1501841 26851216 PMC4761453

[9] Muller MR , Rao A . NFAT, immunity and cancer: a transcription factor comes of age. Nat Rev Immunol. 2010;10:645–56. 10.1038/nri2818 20725108

[10] Bigas A , Espinosa L . The multiple usages of Notch signaling in development, cell differentiation and cancer. Curr Opin Cell Biol. 2018;55:1–7. 10.1016/j.ceb.2018.06.010 30006050

[11] Grabher C , von Boehmer H , Look AT . Notch 1 activation in the molecular pathogenesis of T‐cell acute lymphoblastic leukaemia. Nat Rev Cancer. 2006;6:347–59. 10.1038/nrc1880 16612405

[12] Robles‐Valero J , Lorenzo‐Martin LF , Menacho‐Marquez M , Fernandez‐Pisonero I , Abad A , Camos M , et al. A paradoxical tumor‐suppressor role for the Rac1 exchange factor Vav1 in T cell acute lymphoblastic leukemia. Cancer Cell. 2017;32:608–23.e9. 10.1016/j.ccell.2017.10.004 29136506 PMC5691892

[13] Barreira M , Fabbiano S , Couceiro JR , Torreira E , Martinez‐Torrecuadrada JL , Montoya G , et al. The C‐terminal SH3 domain contributes to the intramolecular inhibition of Vav family proteins. Sci Signal. 2014;7:ra35. 10.1126/scisignal.2004993 24736456

[14] Aghazadeh B , Lowry WE , Huang XY , Rosen MK . Structural basis for relief of autoinhibition of the Dbl homology domain of proto‐oncogene Vav by tyrosine phosphorylation. Cell. 2000;102:625–33. 10.1016/s0092-8674(00)00085-4 11007481

[15] Katzav S , Martin‐Zanca D , Barbacid M . Vav, a novel human oncogene derived from a locus ubiquitously expressed in hematopoietic cells. EMBO J. 1989;8:2283–90. 2477241 10.1002/j.1460-2075.1989.tb08354.x PMC401160

[16] Lopez‐Lago M , Lee H , Cruz C , Movilla N , Bustelo XR . Tyrosine phosphorylation mediates both activation and downmodulation of the biological activity of Vav. Mol Cell Biol. 2000;20:1678–91. 10.1128/mcb.20.5.1678-1691.2000 10669745 PMC85351

[17] Cuadrado M , Robles‐Valero J . VAV proteins as double agents in cancer: oncogenes with tumor suppressor roles. Biology (Basel). 2021;10:888. 10.3390/biology10090888 34571765 PMC8466051

[18] Fernandez‐Zapico ME , Gonzalez‐Paz NC , Weiss E , Savoy DN , Molina JR , Fonseca R , et al. Ectopic expression of VAV1 reveals an unexpected role in pancreatic cancer tumorigenesis. Cancer Cell. 2005;7:39–49. 10.1016/j.ccr.2004.11.024 15652748

[19] Martin H , Mali RS , Ma P , Chatterjee A , Ramdas B , Sims E , et al. Pak and Rac GTPases promote oncogenic KIT‐induced neoplasms. J Clin Invest. 2013;123:4449–63. 10.1172/JCI67509 24091327 PMC3784531

[20] Robles‐Valero J , Lorenzo‐Martin LF , Fernandez‐Pisonero I , Bustelo XR . Rho guanosine nucleotide exchange factors are not such bad guys after all in cancer. Small GTPases. 2019;11:233–9. 10.1080/21541248.2018.1423851 PMC7549630 29313423

[21] Abate F , da Silva‐Almeida AC , Zairis S , Robles‐Valero J , Couronne L , Khiabanian H , et al. Activating mutations and translocations in the guanine exchange factor VAV1 in peripheral T‐cell lymphomas. Proc Natl Acad Sci USA. 2017;114:764–9. 10.1073/pnas.1608839114 28062691 PMC5278460

[22] Boddicker RL , Razidlo GL , Dasari S , Zeng Y , Hu G , Knudson RA , et al. Integrated mate‐pair and RNA sequencing identifies novel, targetable gene fusions in peripheral T‐cell lymphoma. Blood. 2016;128:1234–45. 10.1182/blood-2016-03-707141 27297792 PMC5009513

[23] Crescenzo R , Abate F , Lasorsa E , Tabbo' F , Gaudiano M , Chiesa N , et al. Convergent mutations and kinase fusions lead to oncogenic STAT3 activation in anaplastic large cell lymphoma. Cancer Cell. 2015;27:516–32. 10.1016/j.ccell.2015.03.006 25873174 PMC5898430

[24] Kataoka K , Nagata Y , Kitanaka A , Shiraishi Y , Shimamura T , Yasunaga J , et al. Integrated molecular analysis of adult T cell leukemia/lymphoma. Nat Genet. 2015;47:1304–15. 10.1038/ng.3415 26437031

[25] Park J , Yang J , Wenzel AT , Ramachandran A , Lee WJ , Daniels JC , et al. Genomic analysis of 220 CTCLs identifies a novel recurrent gain‐of‐function alteration in RLTPR (p.Q575E). Blood. 2017;130:1430–40. 10.1182/blood-2017-02-768234 28694326 PMC5609333

[26] Vallois D , Dobay MP , Morin RD , Lemonnier F , Missiaglia E , Juilland M , et al. Activating mutations in genes related to TCR signaling in angioimmunoblastic and other follicular helper T‐cell‐derived lymphomas. Blood. 2016;128:1490–502. 10.1182/blood-2016-02-698977 27369867

[27] Yoo HY , Sung MK , Lee SH , Kim S , Lee H , Park S , et al. A recurrent inactivating mutation in RHOA GTPase in angioimmunoblastic T cell lymphoma. Nat Genet. 2014;46:371–5. 10.1038/ng.2916 24584070

[28] Campbell JD , Alexandrov A , Kim J , Wala J , Berger AH , Pedamallu CS , et al. Distinct patterns of somatic genome alterations in lung adenocarcinomas and squamous cell carcinomas. Nat Genet. 2016;48:607–16. 10.1038/ng.3564 27158780 PMC4884143

[29] Robles‐Valero J , Fernández‐Nevado L , Lorenzo‐Martín LF , Cuadrado M , Fernández‐Pisonero I , Rodríguez‐Fdez S , et al. Cancer‐associated mutations in VAV1 trigger variegated signaling outputs and T‐cell lymphomagenesis. EMBO J. 2021;40:e108125. 10.15252/embj.2021108125 34617326 PMC8591544

[30] Fukumoto K , Sakata‐Yanagimoto M , Fujisawa M , Sakamoto T , Miyoshi H , Suehara Y , et al. VAV1 mutations contribute to development of T‐cell neoplasms in mice. Blood. 2020;136:3018–32. 10.1182/blood.2020006513 32992343 PMC7770564

[31] Casaca A , Nóvoa A , Mallo M . Hoxb6 can interfere with somitogenesis in the posterior embryo through a mechanism independent of its rib‐promoting activity. Development. 2016;143:437–48. 10.1242/dev.133074 26718008

[32] Christophorou MA , Martin‐Zanca D , Soucek L , Lawlor ER , Brown‐Swigart L , Verschuren EW , et al. Temporal dissection of p53 function in vitro and in vivo. Nat Genet. 2005;37:718–26. 10.1038/ng1572 15924142

[33] Jackson EL , Willis N , Mercer K , Bronson RT , Crowley D , Montoya R , et al. Analysis of lung tumor initiation and progression using conditional expression of oncogenic K‐ras. Genes Dev. 2001;15:3243–8. 10.1101/gad.943001 11751630 PMC312845

[34] Lorenzo‐Martín LF , Fernández‐Parejo N , Menacho‐Márquez M , Rodríguez‐Fdez S , Robles‐Valero J , Zumalave S , et al. VAV2 signaling promotes regenerative proliferation in both cutaneous and head and neck squamous cell carcinoma. Nat Commun. 2020;11:4788. 10.1038/s41467-020-18524-3 32963234 PMC7508832

[35] Menacho‐Marquez M , Garcia‐Escudero R , Ojeda V , Abad A , Delgado P , Costa C , et al. The Rho exchange factors Vav2 and Vav3 favor skin tumor initiation and promotion by engaging extracellular signaling loops. PLoS Biol. 2013;11:e1001615. 10.1371/journal.pbio.1001615 23935450 PMC3720258

[36] Sauzeau V , Sevilla MA , Rivas‐Elena JV , de Alava E , Montero MJ , Lopez‐Novoa JM , et al. Vav3 proto‐oncogene deficiency leads to sympathetic hyperactivity and cardiovascular dysfunction. Nat Med. 2006;12:841–5. 10.1038/nm1426 16767097 PMC1997289

[37] Zang S , Li J , Yang H , Zeng H , Han W , Zhang J , et al. Mutations in 5‐methylcytosine oxidase TET2 and RhoA cooperatively disrupt T cell homeostasis. J Clin Invest. 2017;127:2998–3012. 10.1172/JCI92026 28691928 PMC5531410

[38] Mondragón L , Mhaidly R , De Donatis GM , Tosolini M , Dao P , Martin AR , et al. GAPDH overexpression in the T cell lineage promotes angioimmunoblastic T cell lymphoma through an NF‐κB‐dependent mechanism. Cancer Cell. 2019;36:268–287.e210. 10.1016/j.ccell.2019.07.008 31447347

[39] Debackere K , Marcelis L , Demeyer S , Vanden Bempt M , Mentens N , Gielen O , et al. Fusion transcripts FYN‐TRAF3IP2 and KHDRBS1‐LCK hijack T cell receptor signaling in peripheral T‐cell lymphoma, not otherwise specified. Nat Commun. 2021;12:3705. 10.1038/s41467-021-24037-4 34140493 PMC8211700

[40] Wang P , Wang Y , Xie L , Xiao M , Wu J , Xu L , et al. The transcription factor T‐bet is required for optimal type I follicular helper T cell maintenance during acute viral infection. Front Immunol. 2019;10:606. 10.3389/fimmu.2019.00606 30984183 PMC6449430

[41] Turner M , Mee PJ , Walters AE , Quinn ME , Mellor AL , Zamoyska R , et al. A requirement for the Rho‐family GTP exchange factor Vav in positive and negative selection of thymocytes. Immunity. 1997;7:451–60. 10.1016/s1074-7613(00)80367-2 9354466

[42] Heavican TB , Bouska A , Yu J , Lone W , Amador C , Gong Q , et al. Genetic drivers of oncogenic pathways in molecular subgroups of peripheral T‐cell lymphoma. Blood. 2019;133:1664–76. 10.1182/blood-2018-09-872549 30782609 PMC6460420

[43] Imielinski M , Berger AH , Hammerman PS , Hernandez B , Pugh TJ , Hodis E , et al. Mapping the hallmarks of lung adenocarcinoma with massively parallel sequencing. Cell. 2012;150:1107–20. 10.1016/j.cell.2012.08.029 22980975 PMC3557932

[44] Kataoka K , Koya J . Clinical application of genomic aberrations in adult T‐cell leukemia/lymphoma. J Clin Exp Hematop. 2020;60:66–72. 10.3960/jslrt.20019 32779615 PMC7596910

[45] Moon CS , Reglero C , Cortes JR , Quinn SA , Alvarez S , Zhao J , et al. FYN‐TRAF3IP2 induces NF‐κB signaling‐driven peripheral T cell lymphoma. Nat Cancer. 2021;2:98–113. 10.1038/s43018-020-00161-w 33928261 PMC8081346

[46] Rodríguez M , Alonso‐Alonso R , Tomás‐Roca L , Rodriguez Pinilla SM , Manso R , Cereceda L , et al. Peripheral T‐cell lymphoma: molecular profiling recognizes subclasses and identifies prognostic markers. Blood Adv. 2021;5:5588–98. 10.1182/bloodadvances.2021005171 34592752 PMC8714715

[47] Sheikh AA , Cooper L , Feng M , Souza‐Fonseca‐Guimaraes F , Lafouresse F , Duckworth BC , et al. Context‐dependent role for T‐bet in T follicular helper differentiation and germinal center function following viral infection. Cell Rep. 2019;28:1758–72.e1754. 10.1016/j.celrep.2019.07.034 31412245 PMC6711398

[48] Weinstein JS , Lezon‐Geyda K , Maksimova Y , Craft S , Zhang Y , Su M , et al. Global transcriptome analysis and enhancer landscape of human primary T follicular helper and T effector lymphocytes. Blood. 2014;124:3719–29. 10.1182/blood-2014-06-582700 25331115 PMC4263981

[49] DuPage M , Dooley AL , Jacks T . Conditional mouse lung cancer models using adenoviral or lentiviral delivery of Cre recombinase. Nat Protoc. 2009;4:1064–72. 10.1038/nprot.2009.95 19561589 PMC2757265

[50] Hatzi K , Nance JP , Kroenke MA , Bothwell M , Haddad EK , Melnick A , et al. BCL6 orchestrates Tfh cell differentiation via multiple distinct mechanisms. J Exp Med. 2015;212:539–53. 10.1084/jem.20141380 25824819 PMC4387288

[51] Bustelo XR , Rubin SD , Suen KL , Carrasco D , Barbacid M . Developmental expression of the vav protooncogene. Cell Growth Differ. 1993;4:297–308. 8494792

[52] Hornstein I , Pikarsky E , Groysman M , Amir G , Peylan‐Ramu N , Katzav S . The haematopoietic specific signal transducer Vav1 is expressed in a subset of human neuroblastomas. J Pathol. 2003;199:526–33. 10.1002/path.1314 12635144

[53] Lazer G , Idelchuk Y , Schapira V , Pikarsky E , Katzav S . The haematopoietic specific signal transducer Vav1 is aberrantly expressed in lung cancer and plays a role in tumourigenesis. J Pathol. 2009;219:25–34. 10.1002/path.2579 19533802

[54] Fiore D , Cappelli LV , Broccoli A , Zinzani PL , Chan WC , Inghirami G . Peripheral T cell lymphomas: from the bench to the clinic. Nat Rev Cancer. 2020;20:323–42. 10.1038/s41568-020-0247-0 32249838

[55] Mhaidly R , Krug A , Gaulard P , Lemonnier F , Ricci JE , Verhoeyen E . New preclinical models for angioimmunoblastic T‐cell lymphoma: filling the GAP. Oncogenesis. 2020;9:73. 10.1038/s41389-020-00259-x 32796826 PMC7427806

[56] Cortes JR , Ambesi‐Impiombato A , Couronne L , Quinn SA , Kim CS , da Silva Almeida AC , et al. RHOA^G17V^ induces T follicular helper cell specification and promotes lymphomagenesis. Cancer Cell. 2018;33:259–273.e257. 10.1016/j.ccell.2018.01.001 29398449 PMC5811310

[57] de Leval L , Rickman DS , Thielen C , Reynies A , Huang YL , Delsol G , et al. The gene expression profile of nodal peripheral T‐cell lymphoma demonstrates a molecular link between angioimmunoblastic T‐cell lymphoma (AITL) and follicular helper T (TFH) cells. Blood. 2007;109:4952–63. 10.1182/blood-2006-10-055145 17284527

[58] Piccaluga PP , Agostinelli C , Califano A , Carbone A , Fantoni L , Ferrari S , et al. Gene expression analysis of angioimmunoblastic lymphoma indicates derivation from T follicular helper cells and vascular endothelial growth factor deregulation. Cancer Res. 2007;67:10703–10. 10.1158/0008-5472.CAN-07-1708 18006812

[59] Crotty S . T follicular helper cell differentiation, function, and roles in disease. Immunity. 2014;41:529–42. 10.1016/j.immuni.2014.10.004 25367570 PMC4223692

[60] Chiba S , Sakata‐Yanagimoto M . Advances in understanding of angioimmunoblastic T‐cell lymphoma. Leukemia. 2020;34:2592–606. 10.1038/s41375-020-0990-y 32704161 PMC7376827

[61] Salaymeh Y , Farago M , Sebban S , Shalom B , Pikarsky E , Katzav S . Vav1 and mutant K‐Ras synergize in the early development of pancreatic ductal adenocarcinoma in mice. Life Sci Alliance. 2020;3:e202000661. 10.26508/lsa.202000661 32277014 PMC7156281

[62] Shalom B , Farago M , Salaymeh Y , Sebban S , Risling M , Pikarsky E , Katzav S . Vav1 accelerates Ras-driven lung cancer and modulates its tumor microenvironment. Cell Signal. 2022;97:110395. 10.1016/j.cellsig.2022.110395 35752351

