# Supplementary material for: Characterization of the spectrum of trivalent VAV1‐mutation‐driven tumours using a gene‐edited mouse model
Source: Mol Oncol. 2022 Aug 30;16(19):3533–53. doi: 10.1002/1878-0261.13295 (PMC9533688; doi:10.1002/1878-0261.13295)
Supplement: Supplementary file 1 — Fig. S1. Generation of the Vav1 ΔC/ΔC mouse strain. Fig. S2. Vav1 ΔC/ΔC mice show normal lymphoid and myeloid populations. Fig. S3. The activation state of TFH cells is maintained upon reexpression of TP53 in Trp53 ER/ER;Vav1 ΔC/ΔC mice. [file MOL2-16-3533-s001.pdf]

Supplemental Information for

CHARACTERIZATION OF THE SPECTRUM OF TRIVALENT *VAV1* MUTATION-DRIVEN TUMORS USING A GENE-EDITED MOUSE MODEL

by

Javier Robles-Valero *et al*.

This PDF file includes:

Supplementary Figures S1 to S3 and legends

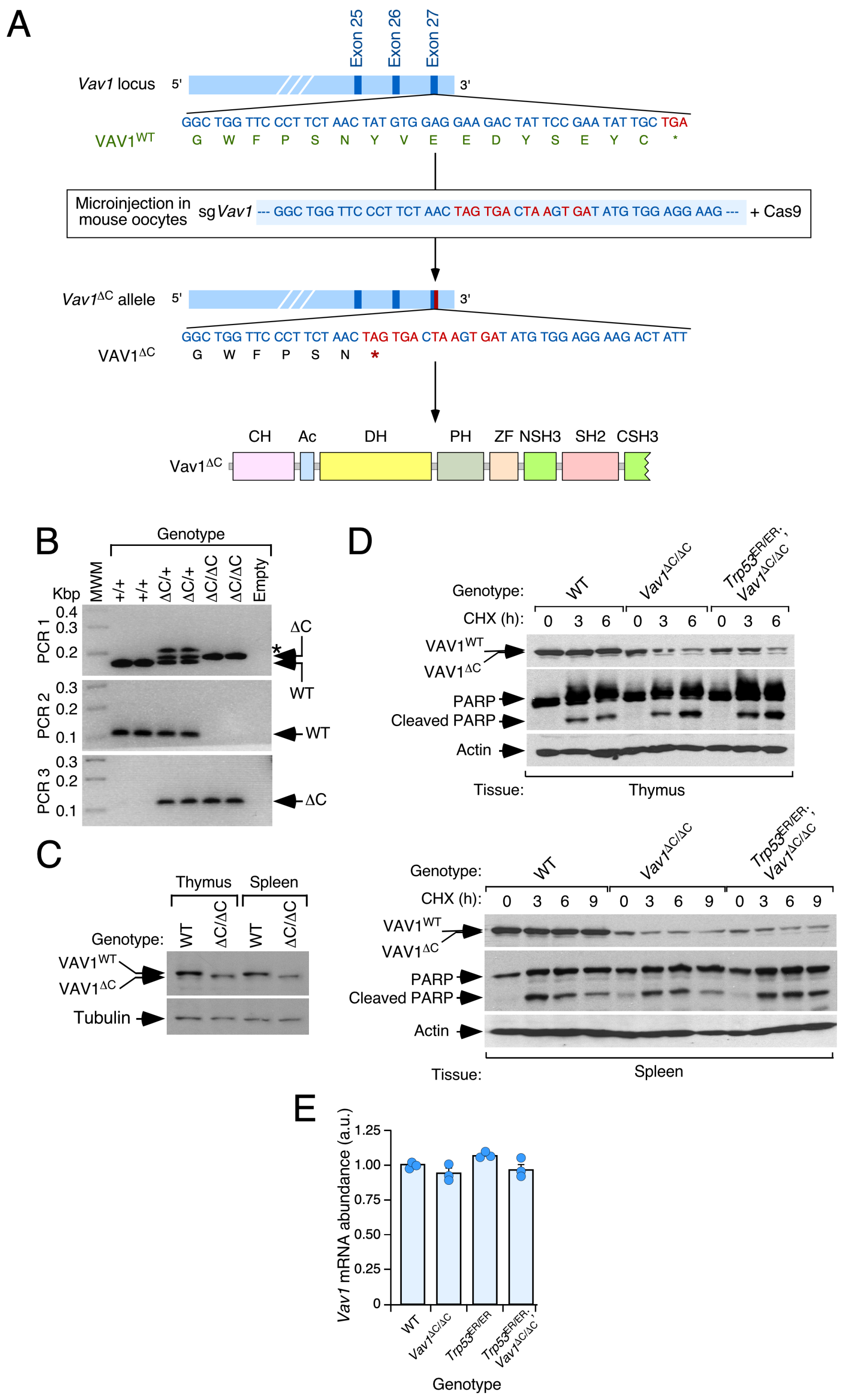

FIGURE S1. Generation of the *Vav1*$^{\Delta C/\Delta C}$ mouse strain

(A) Scheme of the method used to generate the CRISPR-Cas9 gene-edited mouse strain. After the editing step in oocytes, four stop codons (red) are introduced downstream of the Asn^{835} codon, leading to the expression of a C-terminally truncated protein.

(B) Example of the PCR-mediated genotyping of mice of indicated genotypes (top) to identify animals bearing the WT and *Vav1*$^{\Delta C}$ alleles. The asterisk indicates heteroduplexes formed during the last PCR cycles due to the similarity of the PCR products generated from those two alleles. MWM, molecular weight marker.

(C) Representative immunoblot showing the abundance of the WT and mutant protein in total tissue extracts obtained from the thymus and spleen of animals of the indicated genotypes (upper blot). Filters were reblotted with tubulin α to confirm proper loading of samples (bottom blot).

(D) Representative immunoblots showing the abundance of the $VAV1^{WT}$ and $VAV1^{\Delta C}$ protein in total tissue extracts obtained from thymocytes (top) and splenocytes (bottom) from of indicated genotypes that were cultured in the presence of cycloheximide (CHX) at the indicated times. Cycloheximide blocks protein synthesis, so under these conditions we can follow the stability of the indicated VAV1 proteins that were synthesized by cells prior to the cycloheximide treatment. As control, independent filters containing the same tissue extracts were blotted with antibodies to PARP to detect the expected cleaved fragment obtained when cells are treated with cycloheximide. The VAV1 filters were also reblotted with β actin to confirm proper extract loading in each interrogated sample .

(E) qRT-PCR determination of relative abundance *Vav1* mRNA in the indicated genotypes. n = 3 animals per genotype.

In panel E, data represent the mean ± SEM.

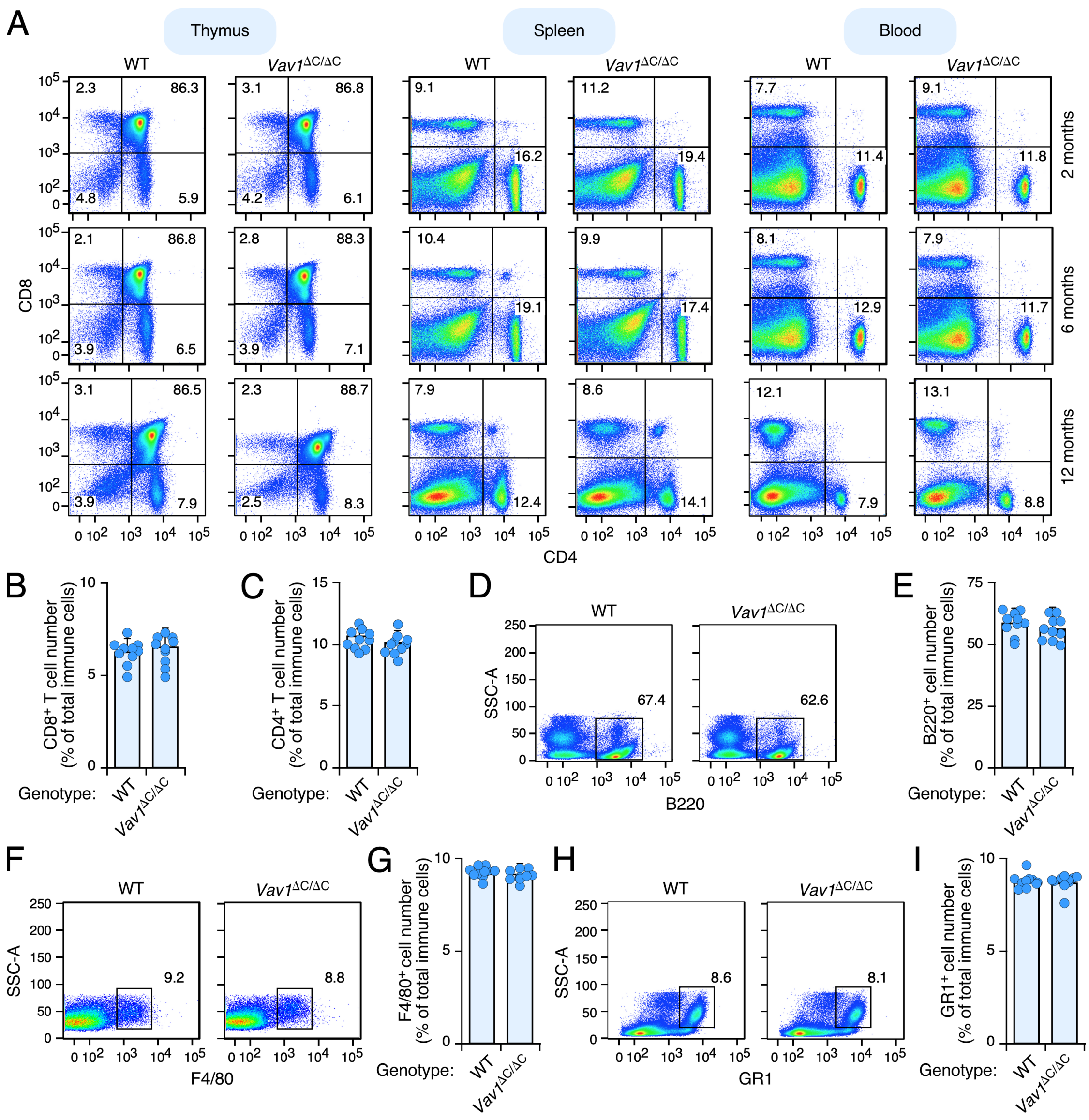

FIGURE S2. ***Vav1***$^{\Delta C/\Delta C}$ **mice show normal lymphoid and myeloid populations**

(A) Flow cytometry analysis of the surface expression of CD8 and CD4 in thymocytes (left panels), splenocytes (middle panels) and blood cells (right panels) from control and *Vav1*$^{\Delta C/\Delta C}$ mice. Animals were analyzed at the ages indicated on the right. In all cases, the numbers indicate the relative percentage (%) of each boxed cell population. n = 10 animals per genotype.

(B and **C)** Quantification of the percentages of CD8^{+} (B) and CD4^{+} (C) T cells in the blood from 6-month-old mice of indicated genotypes. Circles represent values from individual mice. n = 10 mice per genotype.

(D, **F** and **H)** Flow cytometry panels showing percentages of cells staining positive for surface B220 (B cells, D), F4/80 (macrophages, F) and GR1 (granulocytes, H) from 6-month-old control and *Vav1*$^{\Delta C/\Delta C}$ mice. Numbers in each quadrant indicate the relative percentage (%) of each boxed cell population. Similar data were obtained in 10 independent determinations of each genotype.

(E, **G** and **I)** Quantification of the indicated immune cell subtypes from the experiments performed in panels D, F and H, respectively. Circles represent values from individual mice. n = 10 independent determinations/genotype.

Data information: In panels (B, C, E, G, and I), data represent the mean ± SEM.

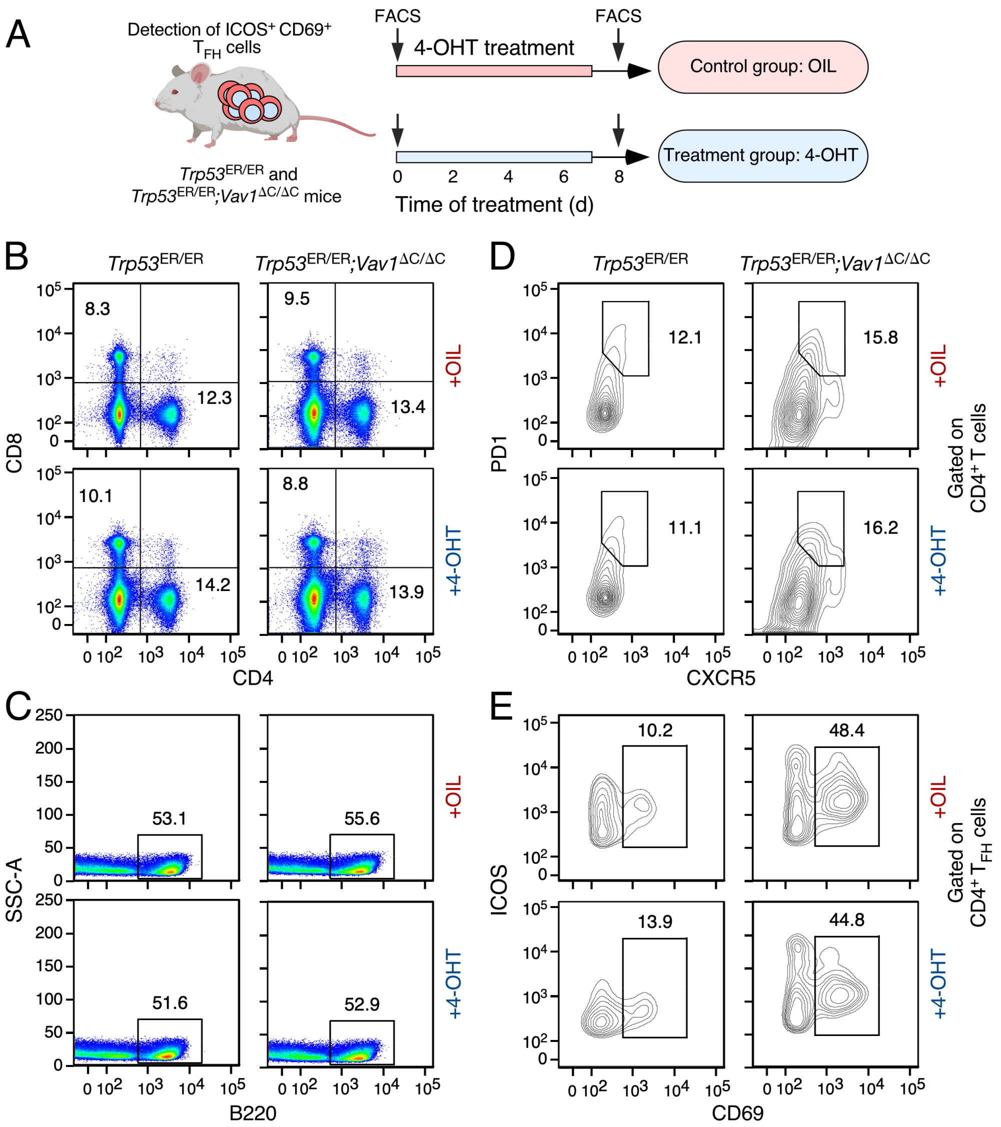

FIGURE S3. The activation state of T$_{FH}$ cells is maintained upon reexpression of TP53 in *Trp53*$^{ER/ER}$;*Vav1*$^{\Delta C/\Delta C}$ mice

(A) Depiction of the experiment used in this figure. See Methods for further details. 4-OHT, 4-hydroxytamoxifen.

(B and **C)** Flow cytometry analysis of the surface expression of CD8 and CD4 (B) and B220 (C) in spleen lymphocytes from five-month-old mice of indicated genotypes (top) and treatment conditions (right). In all cases, the numbers indicate the relative percentage (%) of each boxed cell population. n = 5 animals per genotype.

(D and **E)** Representative flow cytometry plots for the surface expression of PD1 and CXCR5 (D) or ICOS and CD69 (E) in the gated populations (right) of splenic lymphocytes isolated from five-month-old mice of indicated genotypes (top) and treatment conditions (right). Numbers indicate the relative percentage (%) of the cell population selected. n = 5 animals per genotype.
